# Geosmin suppresses defensive behaviour and elicits unusual neural responses in honey bees

**DOI:** 10.1038/s41598-023-30796-5

**Published:** 2023-03-08

**Authors:** Florencia Scarano, Mukilan Deivarajan Suresh, Ettore Tiraboschi, Amélie Cabirol, Morgane Nouvian, Thomas Nowotny, Albrecht Haase

**Affiliations:** 1 Department of Physics, University of Trento, 38120 Trento, Italy; 2 Center for Mind/Brain Sciences (CIMeC), University of Trento, 38068 Rovereto, Italy; 3 Department of Biology, University of Konstanz, 78457 Konstanz, Germany; 4 Zukunftskolleg, University of Konstanz, 78464 Konstanz, Germany; 5 School of Engineering and Informatics, University of Sussex, Brighton, BN1 9QJ UK; 6 Present Address: Department of Fundamental Microbiology, University of Lausanne, CH-1015 Lausanne, Switzerland

**Keywords:** Animal behaviour, Neural circuits, Neurophysiology, Network models

## Abstract

Geosmin is an odorant produced by bacteria in moist soil. It has been found to be extraordinarily relevant to some insects, but the reasons for this are not yet fully understood. Here we report the first tests of the effect of geosmin on honey bees. A stinging assay showed that the defensive behaviour elicited by the bee’s alarm pheromone component isoamyl acetate (IAA) is strongly suppressed by geosmin. Surprisingly, the suppression is, however, only present at very low geosmin concentrations, and disappears at higher concentrations. We investigated the underlying mechanisms at the level of the olfactory receptor neurons by means of electroantennography, finding the responses to mixtures of geosmin and IAA to be lower than to pure IAA, suggesting an interaction of both compounds at the olfactory receptor level. Calcium imaging of the antennal lobe (AL) revealed that neuronal responses to geosmin decreased with increasing concentration, correlating well with the observed behaviour. Computational modelling of odour transduction and coding in the AL suggests that a broader activation of olfactory receptor types by geosmin in combination with lateral inhibition could lead to the observed non-monotonic increasing–decreasing responses to geosmin and thus underlie the specificity of the behavioural response to low geosmin concentrations.

## Introduction

Geosmin is a musty, earthly-smelling compound produced by multiple microorganisms from various clades such as cyanobacteria, actinobacteria (*e.g. Streptomyces sp*.), protozoa, moulds and fungi^1,2^. Actinobacteria (including *Streptomyces*) are widely associated with hymenopteran nests, which they likely protect from pathogens thanks to their natural production of antibiotics^3–5^. A recent study found that fire ant queens (*Solenopsis invicta*) preferentially started new nests in actinobacteria-rich soil. This attraction was mediated in part by geosmin and resulted in a higher survival rate of the queen^6^. Geosmin is also ecologically important for other insects, such as the vinegar fly *Drosophila melanogaster* and the mosquito *Aedes aegypti*. However, it evokes dramatically different responses in those species. Geosmin elicits a strong aversion in *D. melanogaster*, even in the presence of attractive compounds^7,8^. This could be to avoid oviposition on mouldy, unsuitable fruit^8^, or to provide a better contrast with fallen ripe fruits, thus making the search more efficient^9^. In *Ae. aegypti*, on the contrary, geosmin is a strong attractant^10^. This is likely because it signals the presence of wet soil, in which eggs can be laid. Indeed, geosmin is also known for being one of the main components of Petrichor, "the smell of wet soil"^2^.

In the first olfactory processing brain centre of insects, the antennal lobe (AL), most odours elicit a combinatorial pattern of glomerular responses^11,12^. However, some odours, often with high biological relevance (*e.g.* sex pheromones), only activate a single glomerulus. When this response is independently processed also in higher brain centres, resulting in stereotypical behavioural responses, the circuit is termed a labelled line^12^. Geosmin is one of very few compounds that activate only a single glomerulus in *D. melanogaster* and *Ae. Aegypti* mosquitoes^8,10^. In flies, its processing is further functionally segregated in higher brain centres, where it takes priority over other olfactory signals to trigger an avoidance behaviour^8^.

Despite the ecological relevance of geosmin in many insect species, behavioural and physiological responses to this odour have not been previously investigated in the honey bee *Apis mellifera*. Here, we provide the first data to tackle this question. This work was motivated by the question of whether geosmin, which is an olfactory clue of impending summer rain for humans, could also be a weather indicator for honey bees^13^. If so, it should modulate the bee behaviour, as weather-dependent effects on a wide range of bee behaviours have been reported, also for in-hive activities^14^. Behavioural changes in foragers even seem to anticipate changes of the weather^15^.

A possible influence on the defensive behaviour has rarely been investigated. Although there is some evidence that stinging increases under hot and humid conditions^16,17^, the reports of apidologists do not necessarily confirm these observations. We decided to investigate the influence of geosmin on defensive behaviour in this context.

Honey bees defend their colony by stinging potential intruders, a behaviour that is stimulated in the presence of the alarm pheromone released by other colony members^18^. We tested this behaviour in the laboratory using a well-established stinging assay^19^. Furthermore, we searched for neuronal correlates for the behaviour at the level of the olfactory receptor neurons via electroantennography and at the level of the antennal lobe projection neurons via in vivo two-photon calcium imaging^20^ of geosmin-elicited activity. Finally, we used a spiking neural network model to investigate how the observed partially unusual neuronal responses relate to the current understanding of the bee olfactory system.

## Results

### Behavioural responses to geosmin, IAA, and their mixtures

**Figure 1:**
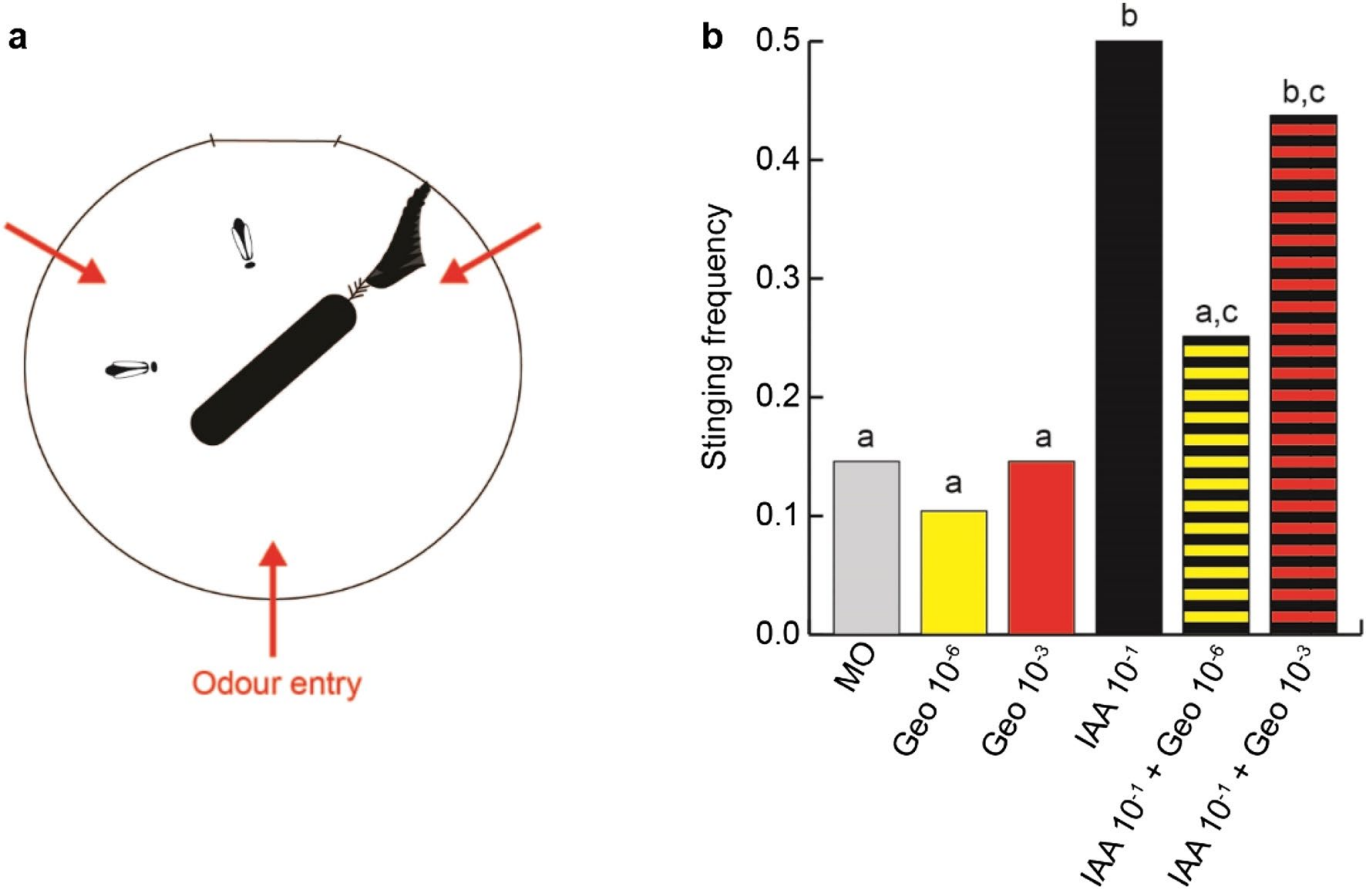
A low concentration of geosmin prevents recruitment into stinging behaviour by IAA. (**a**) Schematic of the behavioural assay. A dyad of bees was presented with a rotating dummy, that they could choose to sting or not. The red arrows denote the entry points of the airflow carrying the odours into the arena. (**b**) Frequency of trials in which at least one of the bees exhibited stinging behaviour (*n* = 48 dyads of bees per group). MO: mineral oil (solvent control); Geo 10^−x^: geosmin at concentration 10^−x^; IAA 10^–1^: isoamyl acetate at concentration 10^–1^. The groups labelled with the same letter are not significantly different from each other (GLM, corrected for multiple comparisons with an FDR procedure).

The stinging behaviour of a dyad of bees towards a black rotating dummy, presented inside an arena was monitored under the exposure to different odour stimuli. In a control group, bees were exposed to pure mineral oil. In this group, in 15% of the trials, at least one of the two bees showed stinging behaviour against the dummy (Fig. 1a**,** Supplementary Movie 1). If bees were exposed to geosmin within the arena, no effect on the frequency of stinging behaviour was observable. If instead bees were exposed to isoamyl acetate (IAA, at a concentration of 10^–1^), an active compound of the bees’ alarm pheromone^21^, the stinging behaviour increased significantly to 50% stinging (*t*(47) = 3.3*, p* = 0.002) as expected (Fig. 1b).

When bees were exposed to a mixture of IAA and a low concentration of geosmin (10^–6^), clear signs of an interaction between both stimuli were observable with a stinging probability of 25%, a significant reduction of the frequency of this behaviour compared with bees exposed to IAA only (*t*(47) = 2.4*, p* = 0.02*)*. There was no significant difference anymore with respect to the mineral oil control (*t*(47) = 1.1, *p* = 0.27, Fig. 1b).

However, when the geosmin concentration in the mixture was 10^–3^, stinging was observed in 40% of the cases, which was not significantly different from the behaviour of the bees stimulated with IAA only (*t*(47) = 0. 6, *p* = 0.57). Indeed, this stinging frequency was again significantly higher than for control bees (*t*(47) = 2.9, *p* = 0.006), suggesting that the behaviour was driven by the response to IAA in this case (Fig. 1b).

### Olfactory receptor neuron responses at the level of the antennae

**Figure 2:**
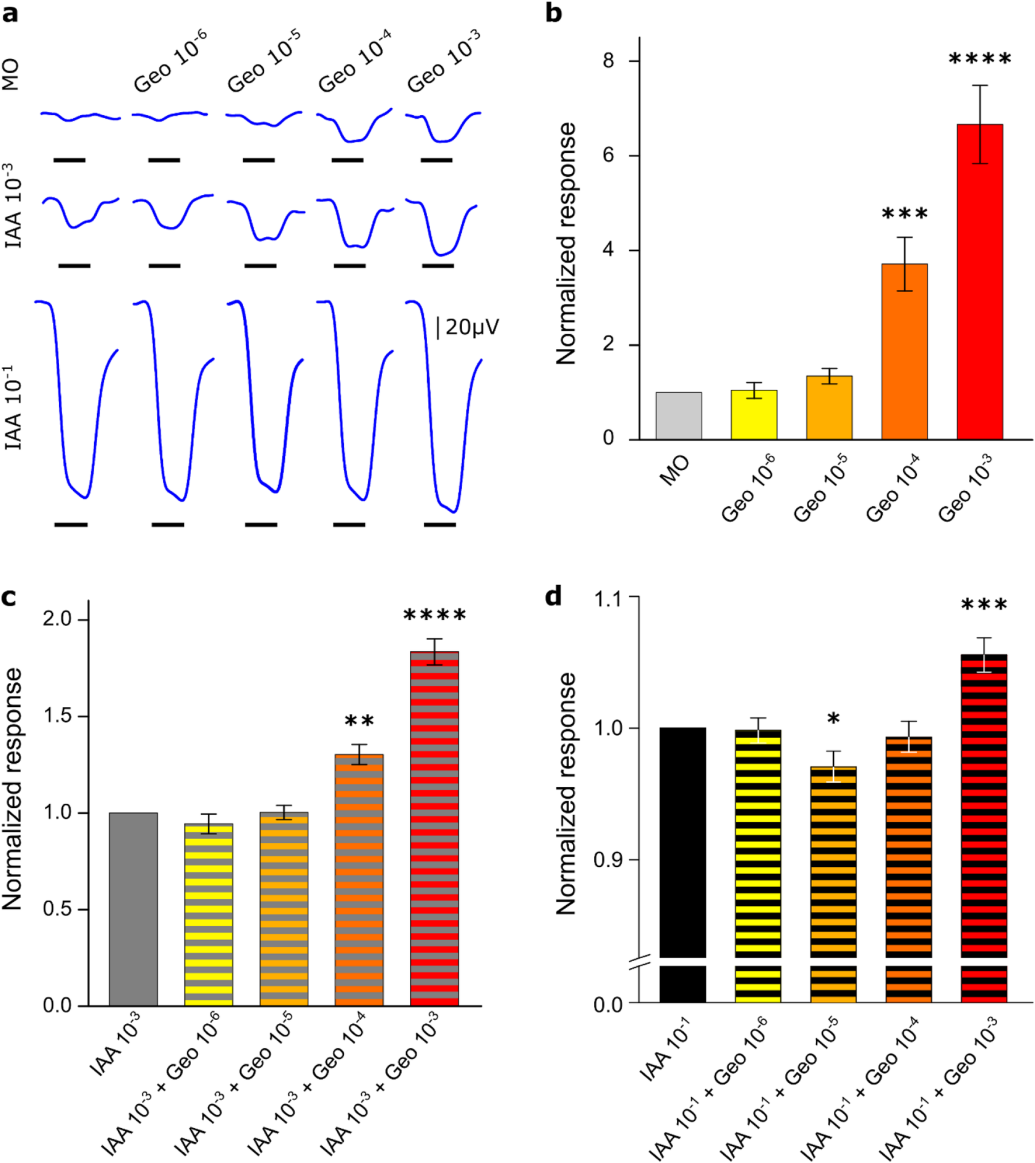
Olfactory receptor neuron responses. (** a**) Example of EAG responses curves from a single bee to all the odours and combinations. The black line below the recording indicates the stimulus presence (2 s). (**b**) Mean of the normalized response amplitude (± SEM) to different concentrations of geosmin. (**c**) Mean of the normalized response amplitude (± SEM) to different concentrations of geosmin combined with IAA 10^–3^. (**d**) Mean of the normalized response amplitude (± SEM) to different concentrations of geosmin combined with IAA 10^–1^. *IAA 10*^*-x*^: Isoamyl acetate 10^-x^ (vol/vol); *Geo 10*^*-x*^: Geosmin 10^-x^ (vol/vol); Statistics are from a Friedman test followed by Dunn’s multiple comparisons with Control, IAA3, and IAA1, in B, C, D respectively: ****p < 0.0001; ***p < 0.001; **p < 0.01; *p < 0.05. Individual amplitude responses from all the tested bees (n = 24) are represented in Supplementary Fig. 1.

We started to search for the neuronal correlates of the behavioural effect of Geosmin combined with IAA at the periphery of the olfactory system, targeting the olfactory receptor neurons (ORNs) in the antennae. Electroantennography measures a voltage change between electrodes at each end of the antenna in response to an odour exposure. This signal, a local field potential recording, has an amplitude proportional to the sum of the activity elicited in all ORNs. In our experiment, honey bee antennae (*n* = 24) were exposed to Geosmin at 4 logarithmically varying concentrations from 10^–6^ to 10^–3^, to two concentrations of IAA: 10^–3^ and 10^–1^, and to mixtures of both odours at all these concentrations. An example of the signals obtained from a single bee in response to the different stimuli is shown in Fig. 2a. However, the magnitude of these signals is impacted by a number of factors: age and status of the animal, conductivity of the electrode, placement of the electrode in proximity to particular ORNs, placement of the ground, and other factors. In order to control for any between-individual differences in the response magnitudes, the response of each bee was first normalized to each concentration of IAA alone (0, 10^–3^, 10^–1^) and then the averages for all the bees were calculated and the statistical analyses were done for repeated measures of EAG responses (Friedman test followed by Dunn’s multiple comparisons). To highlight the statistical differences between the normalized means, we additionally show the raw data for each bee in Supplementary Fig. 1.

Results showed a response significantly different from the mineral oil control at a geosmin concentration of 10^–4^ or higher. The signals followed the typical exponential amplitude increase in response to growing concentrations (Fig. 2b, Supplementary Fig. 1d). The responses to the lowest concentrations of geosmin (10^−5^ and 10^–6^) were not significantly different from the control, probably because the signal difference was below the resolution limit of this method. Yet for a concentration 10^–5^ of geosmin, a clear trend to increase the amplitude can be observed in most of the cases (Fig. 2a,b, Supplementary Fig. 1a,d).

Stimulation with IAA at a concentration of 10^–3^ induced a strong response (Fig. 2a), which seemed reduced in the presence of Geosmin 10^–6^ although the effect is not significant (Fig. 2c, Suppl. Fig. 1b,e). With Geosmin at concentrations 10^–4^ or higher, the signals in response to mixtures were significantly increased in comparison to IAA-only stimulation (Fig. 2c, Supplementary Fig. 1b,e). When the antennae were exposed to mixtures with IAA at a concentration of 10^–1^, the signals showed a reduction when a Geosmin concentration of 10^–5^ was present and in this case, the difference was significant with respect to IAA (*p* = 0.01, Fig. 2d, Supplementary Fig. 1c,f). This difference can be better appreciated in Supplementary Fig. 1c, where individual responses are illustrated. Here, 75% of the bees (18 of 24) display a reduced response in presence of Geosmin at a concentration of 10^–5^ compared to pure IAA at a concentration of 10^–1^ (individuals represented with blue lines).

Results showed that for the behaviourally tested concentration of IAA (10^–1^), the response amplitudes to mixtures were lower than the sum of responses to the individual compounds (green dashed lines in Supplementary Fig. 1f). In the case of 10^–5^ geosmin concentration, we see this suppression reflected in a significant reduction of the response when compared with pure IAA. However, for the behaviourally tested concentration of geosmin, the difference was not significant. This could be in part due to the detectability threshold imposed by the method, *i.e.* stimulation with geosmin alone at the lowest concentrations did not evoke a detectable response signal (Fig. 2b), and in the same way, when combined with IAA 10^–1^ (behavioural tested concentration), geosmin 10^–6^ did not have a significant effect (Fig. 2d). In addition, the observed effect of 10^–5^ concentration of geosmin in the EAG fits with the effect observed for 10^–6^ geosmin concentration in the behavioural responses, suggesting a possible correlation of the ORNs responses with the observed phenomenon at the behavioural level. However, if the behavioural effects were caused already at the receptor level, this interaction should also manifest itself when following signal transduction through to the antennal lobe.

### Projection neuron responses

**Figure 3:**
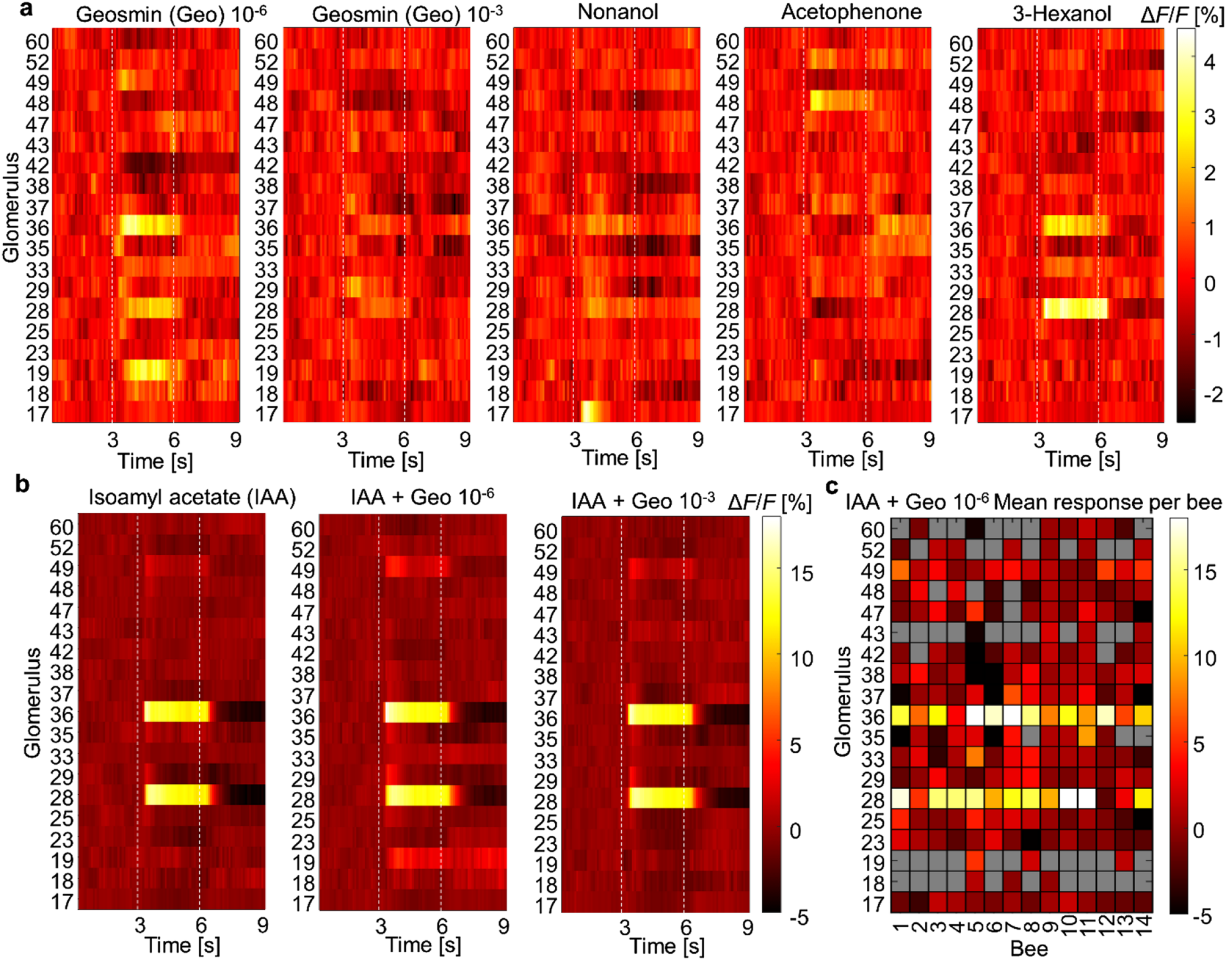
Glomerular responses. (**a**) Mean glomerular response curves to pure Geosmin in two different concentrations, 10^–6^ and 10^–3^, and to three floral odour compounds; 1-nonanol, acetophenone, and 3-hexanol at concentration 5∙10^–3^ for 19 glomeruli (Supplementary Fig. 2a) averaged over all identified glomeruli in 14 bees. Odour stimulation started after 3 s and lasted 3 s, marked by dashed white lines. Colour-coded is the calcium-induced fluorescence change in per cent. Differences between the responses to Geosmin 10^–6^ and 10^–3^ were significant for glomerulus 19 (*t*(2) = -12.1, *p* = 0.013) and glomerulus 36 (*t*(13) = -2.2, *p* = 0.047) (**b**) Mean glomerular response curves to the alarm pheromone compound isoamyl acetate (IAA) at 10^–1^ concentration and to mixtures of IAA with the 2 concentrations of geosmin. The colour scale is different, given the stronger responses to 10^–1^ IAA. Differences between responses to pure IAA and to IAA + Geosmin 10^–6^ are significant for glomerulus 49 (*t*(13) = -2.6, *p* = 0.043) and glomerulus 52 (*t*(6) = -3.3, *p* = 0.02), between IAA and IAA + Geosmin 10^–3^ for glomerulus 43 (*t*(6) = -3.3, *p* = 0.02), and between IAA + Geosmin 10^–6^ and IAA + Geosmin 10^–3^ for glomerulus 52 (*t*(6) = 3.2, *p* = 0.028). (**c**) Time-averaged glomerular response amplitudes to the mixture of IAA and 10^–6^ geosmin in each bee, showing the stereotypy of the response. Grey represents cases in which glomeruli were not clearly identified.

To trace the odour-induced activity across the next processing level, we performed calcium imaging of the projection neurons in the honey bee antennal lobes. These neurons convey information from the ALs to higher-order brain centres. Imaging experiments were performed on 14 bees exposed to the same odours at the same concentrations as in the behavioural assay. The change in fluorescence induced by the odour stimulus was recorded in 19 glomeruli (Supplementary Fig. 2). When averaged over the 3 s stimulus period, these glomerular signals showed highly stereotypical response patterns across bees (Fig. 3c shows the IAA geosmin mixture as an example).

The average temporal response curves show the highly different responses to 4 pure odours (Fig. 3a): Geosmin at the same two concentrations as used in the behavioural tests, 10^–6^ and 10^–3^ in mineral oil, and the three floral odours 1-nonanol, acetophenone, and 3-hexanol all at a concentration of 5∙10^–3^ in mineral oil. Geosmin at concentration 10^–6^ elicits responses in various glomeruli, the spectrum turns out to be broader than those of the floral odour. It shows tonic excitatory responses in 3 glomeruli (19, 28, 36), phasic excitatory responses in other 4 (25, 29, 35, 49), and inhibition in at least 3 glomeruli (42, 48, 60), while the floral odours elicit comparable responses in only up to 4 glomeruli. Surprisingly, the responses to geosmin disappear almost completely at the 1000-fold higher concentration of 10^–3^, suggesting a non-monotonic concentration dependence in the projection neuron (PN) response pattern. Differences between Geosmin 10^–6^ and 10^–3^ are significant for glomerulus 19 (*t*(2) = − 12.1, *p* = 0.013) and glomerulus 36 (*t*(13) = − 2.2, *p* = 0.047). The mean values suggest a difference also in glomerulus 28, but because of the high fluctuation across subjects, the effect is not significant (*t*(13) = − 1.5, *p* = 0.17).

Next, bees were stimulated with the alarm pheromone compound IAA again at the same concentration as in the behavioural experiment of 10^–1^, which is at least 20-fold higher than the other stimuli and accordingly elicits much stronger responses. In addition to IAA, also its mixtures with geosmin were tested. Noticeably, all glomeruli responding to IAA, seem also to be responding to geosmin. Also in the mixtures, the geosmin contributions are clearly visible at a concentration of 10^–6^ but almost completely disappear at a concentration of 10^–3^. Differences between pure IAA and IAA + Geosmin 10^–6^ are significant for glomerulus 49 (*t*(13) = -2.6, *p* = 0.043) and glomerulus 52 (*t*(6) = − 3.3, *p* = 0.02). Mean values suggest a difference also in glomerulus 19, but the glomerulus is clearly identified only in three bees (Fig. 3c), so the effect is not significant (*t*(2) = − 2.5, *p* = 0.14). These differences disappear for the mixture IAA + Geosmin 10^–3^. Response to this mixture is significantly different from response to pure IAA for glomerulus 43 (*t*(6) = − 3.3, *p* = 0.02). Responses to IAA + Geosmin 10^–6^ and IAA + Geosmin 10^–3^ differ significantly for glomerulus 52 (*t*(6) = 3.2, *p* = 0.028).

A significant interaction between geosmin and IAA was not detectable and responses to the mixture seem to be accurately the sum of the single compound responses. Therefore, we could not directly confirm the interaction between the two odours at the level of the antennae or in the AL. However, we also could not exclude such interactions since only 12% of all glomeruli were optically accessible. Considering only the measured PN signals, the evidence so far points towards interactions between IAA and geosmin occurring only in higher-level brain centres such as the mushroom body (MB) or the lateral horn (LH).

### Computational modelling

**Figure 4:**
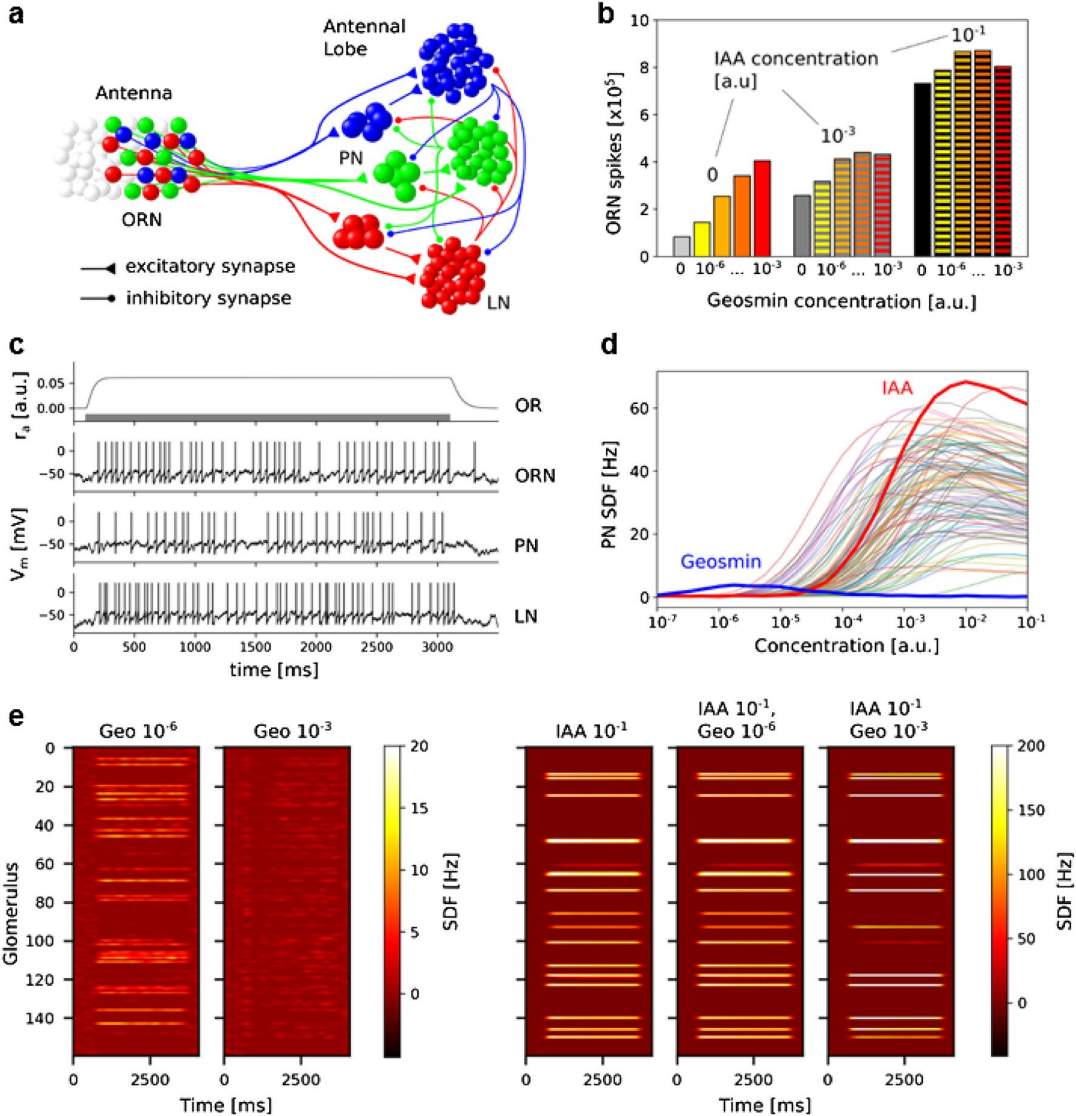
Computational Model. (**a**) Circuit diagram of the model. ORNs excite PNs and LNs in the AL according to their receptor type (colours). LNs also receive excitation from the PNs within the same glomerulus. LNs inhibit PNs and LNs in all other glomeruli but not in their own. (**b**) Response of the ORNs to different mixtures of the “IAA” and “Geosmin” odours. (**c**) Example data for a typical odour response. The bar in the top panel indicates odour exposure. For ORNs, PNs and LNs, the trace of one arbitrary example neuron connected to the most strongly responding glomerulus is shown. Spikes were added to the LIF membrane potential traces as vertical lines for clarity.** (d**) Time-averaged responses of the PNs (Spike density function SDF) in the strongest responding glomerulus in response to 100 different simulated odours presented for 3 s each and at different concentrations. (**e**) Heat maps illustrating the mean PN activity across each of the glomeruli in response to “Geosmin”, “IAA”, and mixtures of the two odours.

While it is well-known that responses of PNs as a function of time are complex and non-monotonic and the response of individual PNs as a function of concentration can go up as well as down^22^, it seems unusual that phasic responses to geosmin in all observed glomeruli appear to diminish for higher concentrations and practically disappear for a concentration of 10^–3^. We have built a computational spiking neural network model of the early olfactory system of bees in order to investigate whether this and our other observations are consistent with our current understanding of the system. The model builds on earlier works^23,24^ and describes olfactory receptors with a two-stage binding and activation process^25^. Olfactory receptors then excite olfactory receptor neurons (ORN), which in turn excite projection neurons (PNs) and local neurons (LNs) in the antennal lobe. All ORNs with the same receptor type project to the same glomerulus^26^. LNs inhibit PNs and LNs in all other glomeruli. The circuit is illustrated in Fig. 4a. The model has 160 receptor types and hence glomeruli, and 5 PNs and 25 LNs per glomerulus, reflecting the current best estimates. We simulated 60 ORNs of each type, meaning that each simulated ORN represents about 10 ORN in reality. Further details are given in the Methods. Figure 4c shows an example data trace from the simulations. Odours were introduced as a step change from zero to a constant concentration for 3 s (grey bar) and then set back to 0. OR activation commences immediately upon odour onset and then leads to spiking in ORNs, followed with barely noticeable delay by PN and LN spikes. One can make out a hint of the spike rate adaptation in the example ORN and LN but this becomes more pronounced for higher spike rates. PNs do not have spike rate adaptation in this model.

By inspecting the EAG recordings and PN imaging results we hypothesized that the unusual declining responses in all observed glomeruli for higher concentrations of Geosmin could be due to the local inhibition mediated by LNs in the AL. Furthermore, non-monotonic behaviour has not been widely reported so we reasoned that Geosmin must have a particular property that makes it susceptible to excess inhibition. We explored the properties of odour responses, including the sensitivity (*η* in the OR model), breadth of the response across receptor types (*σ* in the OR model) and the activation (*k*_*2*_ in the OR model). We found that the breadth of the response was the decisive factor leading to non-monotonic response-concentration relationships in the PN. Figure 4d illustrates this result. We generated 98 odours with a random distribution of response properties and two with more specific ones, one odour that had a very broad response profile, which we identify with Geosmin, and one odour that had an average width profile but very high activation that we identify with IAA. As can be seen in Fig. 4d, “Geosmin” shows a non-monotonic response as a function of concentration while “IAA” is essentially monotonic. The random sample of other odours differ in their behaviour but have in the majority typical, increasing sigmoid response curves. We found that the monotonicity (see “Methods” for a formal definition) or lack thereof of odour responses strongly correlates with the width of the odour profile (Supplementary Figs. 3, 4, 5, 6). The intuition behind the non-monotonic behaviour for odours with unusually broad responses is that with increasing concentration, more OR types are activated, increasing the number of involved glomeruli and hence the global inhibition in addition to the increase of inhibition due to increased activation of OR types which are already active. At the same time, the excitation to each glomerulus increases only according to the sigmoid response curve of the corresponding OR type. When the response profile is broad, the combined increases of inhibition can outweigh the increase in excitation and the overall PN response decreases. For narrower response profiles, the excess inhibition from newly recruited OR types is less and the PN response continues to increase.

We then asked whether we can also reproduce the observed interactions of geosmin and IAA seen in the EAG data (Fig. 2). Here, we interpreted the total number of ORN spikes across all OR types as a sensible proxy of an EAG measurement and reasoned that interactions are likely to be due to syntopic mixture effects at the receptors. With this in mind, Geosmin would lead to inhibition of IAA responses on the antenna if its activation was lower than IAA’s. We, therefore, set the activation rate *k*_2_ of “Geosmin” lower than of “IAA” and generated ORN spike counts for mixtures of “Geosmin” and “IAA”. Through manual exploration, we found that for a roughly 1:3 relationship (*k*_2_^Geo^ = 0.03 kHz, *k*_2_^IAA^ = 0.1 kHz) the responses exhibited suppression effects that showed commonalities with the experimental observations (Figs. 2b–d, 4b). The exact quantitative relationship at different concentration ratios depends, besides on the *k*_2_ ratio, also on the overlap of “Geosmin” and “IAA” OR activation profiles. We tested this by generating “Geosmin” and “IAA” as Gaussian response profiles with a specific distance between their maxima. For small distances (large overlap), the syntopic suppression of IAA responses dominates, and for large distances (small overlap) the responses are more additive (Supplementary Fig. 7). While the model does reproduce suppression of IAA responses by geosmin in principle, we were not able to fully reproduce the more or less constant responses seen in Fig. 2d with this scheme and without losing the match of model behaviour and experiments on the level of the AL (see above). It is, however, likely that with other ways of generating overlapping response profiles or, indeed, other than Gaussian response profiles, the experimental observations could be replicated more closely.

Finally, we asked whether the “Geosmin” and “IAA” odours generated to reproduce the non-monotonic behaviour in PNs and the sub-additive response properties in the EAG would produce PN responses that are similar to the calcium imaging data of PN activity in the AL. Figure 4e shows the simulated response patterns of the PNs in response to Geosmin at 10^–6^, 10^–3^ (compare Fig. 3a), IAA at 10^–1^, and the mixtures of IAA at 10^–1^ and Geosmin at 10^–6^ and 10^–3^ (compare Fig. 3b). Overall, the responses look similar to the experimental data with a moderate response to “Geosmin” 10^–6^, which essentially disappears at 10^–3^. In the mixtures, adding “Geosmin” 10^–6^ to IAA 10^–1^ has almost no visible effects while adding “Geosmin” 10^–3^ appears to sharpen the response profile somewhat, where glomeruli strongly activated by “IAA” and “geosmin” get even more activated, and weakly activated glomeruli are slightly depressed, presumably due to added global inhibition by the LNs. By visual inspection, similar effects appear to be present in the experimental data (Fig. 3b) but we did not identify statistically significant effects.

## Discussion

### Behavioural effects

While defending the colony is of critical importance for honeybees, it is also very costly. Their barbed stinger remains embedded in elastic materials and tears off from the abdomen when the bee pulls away. Thus, the decision to respond to the alarm pheromone by stinging should be tightly regulated to avoid unnecessary losses in the workforce. There are several proofs that this is indeed the case: alarm pheromone responsiveness decreases when its concentration is too high^27^, when group size increases^28^, and in the presence of appetitive compounds^19^. These additional cues may indicate to bees that the threat is already being taken care of, or that this is not the right context for a defensive response. Here, we report yet another instance of such a suppression of alarm pheromone response, elicited by the presence of geosmin (Fig. 1). Intriguingly, we tested the bees with 2 concentrations of geosmin and only found this inhibiting effect when using the lowest one. Concentration-dependent effects of odours on behaviour have been reported before, for example, weaver ants exhibit a series of behaviours as they approach (and thus perceive an increase in concentration) a source releasing 1-hexanol, a component of their own alarm pheromone^29^. Recently, non-monotonic behavioural responses to IAA itself have been reported in honey bees^27^. More precisely, the stinging frequency of individual bees tested in the same behavioural assay increased with IAA concentration up to 2.5∙10^–1^, but then dropped back when higher concentrations were used. Added to the strong overlap observed between the glomeruli activated by geosmin and IAA, one hypothesis could be that additive activation by geosmin would shift the behavioural response towards the declining range of this IAA dose–response curve. While this hypothesis needs to be further tested, we find it unlikely since the decrease in stinging behaviour only appears at very high IAA concentrations, presumably requiring much stronger activations than the ones recorded here.

A similar modulation of the defensive behaviour, whereby stinging responsiveness to the alarm pheromone was reduced by the addition of another odour, was observed previously^19^. This effect was specific to floral compounds with an appetitive value. Does geosmin signal food for honey bees? This hypothesis could be tested by measuring the frequency of spontaneous extension of the proboscis in response to this compound in a future study^19^. *Streptomyces* bacteria have been found in flowers and can protect honeybees against pathogens^30^. While the vast majority of *Streptomyces* species can produce geosmin^31^, actual detection of geosmin within the floral bouquet has however only been reported in a few cacti species^32^. Whether this compound could attract bees to flowers and thus directly participate in mediating mutual interactions thus remains to be verified. Another exciting possibility would be that geosmin, while not present in flowers specifically, signals a general increase in nectar availability: indeed nectar production increases after rain^33^ when geosmin is also released by soil bacteria. The presence of geosmin could thus trigger foraging responses in honey bees, just like appetitive floral compounds.

Geosmin is an ecologically relevant compound eliciting either attraction or repulsion in a number of arthropod species including flies^7,8^, mosquitoes^5^ and springtails^34^. Although its ecological function remains mysterious in the case of honey bees, our data suggest that it may be equally relevant for this important pollinator, and worth investigating further.

### ORN responses

Electroantennography results suggest that the non-monotonic concentration dependence of the behaviour does not stem from an anomalous sensitivity of receptors to geosmin, as the EAG amplitude shows a typical increasing dose–response curve for stimuli with the pure odour (Fig. 2b). However, since a behavioural effect was observed only to mixtures between geosmin and IAA, most interesting are potential interactions between these two stimuli. Indeed, compared to an expectable linear sum of the pure odour response amplitudes^35^, the response amplitude to the binary mixture is not showing this clear increase with concentration. The mixture of geosmin with a low IAA concentration shows a tendency of a reduced signal increase with increasing geosmin concentration (Fig. 2c). For the high IAA concentration (Fig. 2d), the effect becomes significant: the addition of geosmin 10^–5^ causes a significant decrease in the signal, and the expected significant increase is not observable until a geosmin concentration of 10^–3^. Dose–response curves in which a higher concentration does not necessarily produce an increase in the response amplitude, have been observed previously in EAG measurements in honey bees, and these were also responses to odour mixtures^36^.

An interaction of geosmin and IAA is in line with the behavioural signal, where the effect of geosmin on IAA-induced stinging was significant for geosmin 10^–6^ but vanished for geosmin 10^–3^.

The underlying mechanism for this interaction could be the masking of IAA by geosmin at the level of the receptors, an effect that has been reported^37^. Interestingly, in this work it was IAA that had been identified as a strong masking agent but was also shown to be maskable by some odours^37^. One requirement for masking is the sensitivity of receptors to both odours. The EAG signal being the sum of the activity in a large part of the 60.000 ORNs, does not allow to clearly identify masking mechanisms limited to a small fraction of ORNs. To gain further insights into potential interactions, we performed calcium imaging of the odour responses at the level of the antennal lobe.

### PN responses

One requirement for an interaction between both odours was confirmed by the response maps of the glomerular projection neurons. Although only a subset of 19 out of 160 glomeruli (Supplementary Fig. 1) could be consistently identified in various subjects, all the glomeruli that were activated by IAA were found to be also activated by geosmin 10^–6^ (Fig. 3).

A further surprising finding of the calcium imaging was a non-monotonic dependence of the projection neuron responses to the geosmin concentration (Fig. 3a,b). Using the same concentrations as in the behavioural studies, the broad and strong responses to geosmin 10^–6^ almost completely disappeared for 10^–3^. This again is in line with the behavioural data. However, such a non-monotonic concentration dependence seems to be very rare. At the level of the antennal lobe, it was not reported in bees before. A work in moths shows such responses to pheromone components from electrophysiological recordings in the AL, the type of neurons however remains undetermined^38^. At the level of the honey bee mushroom body input, PN boutons showed highly varying concentration dependencies^39^, monotonic increases and decreases as well as non-monotonic changes. The authors suggested inhibition at the level of the boutons. Another work in moths found only monotonic increases at the level of the AL in the PN dendrites but at the level of the PN somata also non-monotonic responses^40^. The suggested underlying mechanism was postsynaptic inhibition. In addition to those two positions at which inhibition could create such a change in concentration dependence, our data now suggest that also inhibition within the antennal lobe might have an effect.

Imaging the response to mixtures of IAA and geosmin confirmed the non-monotonic dependence on the geosmin concentration also under this condition. However, an interaction of IAA and geosmin was not observed in the imaged subset of glomeruli, the mixtures elicited a response that appears to be the exact sum of the pure odour responses of both compounds (Fig. 3b). This suggests two possible scenarios: either interactions vary across glomeruli and are not visible in the subset we were able to image, or our observations on the EAG signal are not due to an interaction of ORNs, and neither do interactions happen at the level of the antennal lobe where odours are identified, but rather in the higher brain centres such as the mushroom body and the lateral horn where the valence of the odours is extracted^41^.

A final important result of the calcium imaging is the observation of a broad response map that geosmin elicits, which proves combinatorial coding of this odour in contrast to the results in fruit flies^8^ and mosquitoes^10^ where the response was limited to a single glomerulus, which corresponds to the complementary coding mechanism of a labelled line^42^. Studies of the evolution of olfactory receptors indeed suggest that a variation in coding modes is likely between species^43^. The width of the spectrum of receptors that geosmin activated seems indeed broader than those of the floral odours that were imaged in comparison (Fig. 3a). This observation proved crucial for the following attempt to investigate how the inverted concentration dependence might arise from the processing of the regular input signals in the antennal lobe network.

### Neuronal network model

The puzzling results of an inverse concentration dependence of the geosmin response in the antennal lobe output neurons, as well as the interaction between geosmin and IAA at the level of the olfactory receptor neurons, were contextualised with the help of a computational model of the olfactory receptor neurons and the AL. We have demonstrated that such a response pattern can arise as a result of the specific properties of the receptor responses and a specific balance of excitatory-inhibitory coupling in the AL. The persistence of some weak phasic responses at the higher geosmin concentration suggests that inhibition rather than the disappearance of the excitatory drive is responsible for the signal change. This is reproduced by the model in which weak phasic responses remain at higher concentrations because the inhibition of PNs from local interneurons always arrives slightly later than the direct excitation from sensory neurons. The almost vanishing geosmin response for larger concentrations may at first glance be surprising as at the same time other odorants have perfectly normal response profiles with increasing or saturating responses with increasing concentration and the pattern of inhibitory connections is of course a circuit property, independent of the odour. With our computational model we have shown that contrary to intuitive expectations, the same inhibitory circuit can lead to qualitatively different response behaviours based on the breadth, sensitivity and activation of the ensemble of ORNs in response to different odorants. As illustrated in Supplementary Figs. 3, 4, 5, depending on the breadth of activation across the receptor reservoir, PN response profiles as a function of increasing concentration can flip from monotonically increasing to increasing–decreasing, and, in the extreme case, vanishing. This result and the observed unusual concentration tuning of the geosmin response suggest that in contrast to other insects, geosmin responses in bees are indeed broader compared to other odours.

### Locations of mixture interactions

The literature suggests that odour interactions occur at different levels of the olfactory system. At the receptor level, odorants can elicit inhibitive responses. Mixed with an excitatory component, a suppression of the latter has been observed^37^. This alters not only the amplitude of the ORN response but also the response dynamics, which likely affects not only the perception of the odour intensity but also the perception of odour identity^44^. These effects are specific to individual receptors and would show up in an EAG signal as a small reduction of the summed neuronal response to a mixture compared to the sum of the responses to the individual components, as observed in our experiments.

At the projection neuron level, the effect of mixture suppression is commonly observed, *i.e.* the glomerular response to a mixture is lower than that to its strongest component^45^. In honey bees, the two distinct AL pathways were found to respond differently to odour mixtures. While the medial-tract projection neurons (m-PNs) were dominated by the most effective compound, the lateral-tract projection neurons (l-PNs), which were imaged in our experiments, often showed suppressed responses to mixtures^46^. The role of the local inhibitory interneuron in this interaction was demonstrated in *Drosophila* by the administration of picrotoxin, an antagonist of GABA_A_-like receptors, which eliminated mixture suppression at the PN level^47^. Computational modelling confirmed that overshadowing and blocking of one odour response by another likely results from lateral inhibition in the honey bee AL^48^. Simulations also suggest that mixtures allow faster and more reliable olfactory coding, which could be one of the reasons why animals often use mixtures in chemical signalling^24^.

In moths, also complementary effects were observed. A bioactive mixture strongly activated an additional glomerulus, insensitive to the single components^49^. Whether this synergetic effect occurs at the level of the receptors or the AL network remains an open question.

Other studies have shown that the processing of mixture-related information in the AL is not uniform across glomeruli, which could offer a possible explanation for the lack of evidence in our PN data, and suggest that a final integration of the information on the mixture may happen in higher-order brain centres^50^.

The role of odour valence in mixture interactions was studied in *Drosophila*. Mixtures of odours with opposing valences elicited strong inhibition in attractant-responsive glomeruli. Manipulation of single ORNs by silencing and optogenetic activation revealed glomerulus-specific crosstalk between the attractant- and repellent-responsive circuits. This selective lateral inhibition was found to be crucial in the processing of conflicting sensory information^51^.

Taken together, these works paint a similar picture to the one we are gaining from our data, namely that the interaction within odour mixtures does not have a specific interaction point, but is an effect that is generated, amplified, and deciphered along different processing stages in the insect's olfactory system.

### Compatibility of the individual results

Behavioural experiments showed that the stinging behaviour elicited by IAA is strongly reduced by Geosmin, but only at a low concentration of 10^–6^, at an increased concentration of 10^–3^ the effect entirely disappears. A test for the interaction between both odours at the level of the antennae shows a compatible result: we see a significant reduction of the overall response signal to the IAA + Geo 10^–5^ with respect to pure IAA, at increased geosmin concentrations the effect vanishes. The fact that the behavioural effect and PN responses are detected already at geosmin concentrations of 10^–6^, for which the antennae did not show a measurable EAG, shows how difficult it is to compare concentrations across experiments^52^. Concentrations can only be measured in terms of dilutions of the odour source, as the amounts of geosmin arriving at the antennae are far below the detection limit of any device. Because the distribution of odours in the experimental setups for in vivo imaging and EAG measurements are very different, comparable scenarios are indicated not by the same dilution but rather by similar effect sizes. While a concentration of 10^–6^ geosmin does not elicit an EAG signal above threshold, it evokes clear responses in calcium imaging and behaviour, suggesting a lower "effective" concentration in EAG.

The calcium imaging experiments shed light on the odour responses of geosmin, IAA, and the mixtures at the level of the antennal lobe output neurons. The most surprising result here is that also geosmin by itself shows a reduced response in the imaged glomeruli when concentration is increased, which supports the observation of reduced behavioural responses but seems to contradict the EAG results, where ORN responses increased with concentration over the observed range of concentrations. However, the computer model offers an explanation, namely that despite a monotonic increase in ORN responses to geosmin, lateral inhibition in the AL could cause the PN signal to be strongly reduced as observed by calcium imaging.

One inconsistency remains, however: at the level of the antennal lobe, no interaction between geosmin and IAA could be observed, the mixture signal is almost exactly the sum of the signals of both components. The presence of such an interaction is suggested by both the EAG and the behavioural experiment. However, our PN activity maps also do not rule out the existence of such interaction at the level of the antennal lobe, as our imaging technique allowed us to record from 19 clearly identifiable glomeruli out of the approximately 160 present in a honey bee antennal lobe. Of these 19 glomeruli, only 2 responded strongly to IAA. Therefore, it is not unlikely that an interaction could be present in glomeruli that are not accessible to optical imaging. However, since there is no direct evidence for an interaction, there remains another level at which the interaction could take place, namely in higher-level brain centres. To date, imaging of response patterns in the honey bee mushroom body^53^ and the lateral horn^54^ has proven extremely challenging and the coding of odour mixtures has never been studied there. But the recent development of a transgenic honey bee line, expressing a pan-neuronal calcium sensor, raises hope that in the future even parallel imaging of the entire olfactory system would be possible and allow following mixture interactions along all processing steps. Imaging studies in *Drosophila* suggest that modulation of a behaviour triggered by the alarm pheromone may well occur there, as coding for odour valence^55,56^ and the segregation of pheromones from food odours^57^ have been observed in these parts of the brain.

A further result of the calcium imaging experiment was that, opposite to flies and mosquitoes, in bees geosmin is not coded by a labelled line, but its response spectrum seems on contrary even larger than those of the tested floral and pheromonal compounds. The computational model showed that the non-monotonic concentration dependence of the geosmin response and its broad response spectrum might indeed be linked to non-monotonic response amplitude due to lateral inhibition. The model indeed also provides an example configuration in which the IAA signal in the antenna is reduced as geosmin is added, due to syntopic interactions of the odours at the receptors. This does not fully reproduce the detailed results from the behaviour and EAG, in which the strongest suppression was observed at low geosmin concentrations. The total response of the receptor reservoir to mixtures of IAA and geosmin depends on the details of the affinities of all receptor types to both odours and their concentration dependence, including syntopic interactions, but also recruitment of more receptors and more receptor types with increasing concentration, which may well account for the differences observed.

### Outlook and conclusions

These results constitute another step towards understanding the olfactory modulation of defensive behaviour in honey bees, both at the behavioural and neuronal levels. Besides a fundamental understanding of the neuronal circuits leading to stinging, this may also be of practical use for beekeeping.

Recent studies proposed the use of geosmin as a natural repellent against pest insects such as *D. suzukii*^58^. Further investigation on the consequences of geosmin on non-target insects such as honey bees should be performed before any commercialization of geosmin for this purpose.

Context is key for pheromone responses, hence in-field experiments will be required to fully understand the effect of geosmin. This should include monitoring behaviour, especially at the hive entrance, controlling the bees' age, their role in the colony, and whether they fly out or return.

Following the elicited neuronal activity into the higher-order olfactory processing centres such as the lateral horn and mushroom bodies would also be a valuable future direction in order to study the valence of geosmin, IAA, and its mixtures at different concentrations. This might be complemented with additional modelling to better underpin our understanding of underlying circuits and processes and generate testable hypotheses to challenge that understanding.

Taken together, our data and model provide the first evidence that geosmin strongly modulates the defensive behaviour of honey bees based on unusual neuronal response properties; an ecological relevance for bees is therefore likely. The nature of this relevance remains purely speculative. For humans, geosmin serves as a weather indicator. The mechanism why the smell of geosmin often precedes the rain has recently been revealed^13^: when raindrops hit the ground, they produce aerosols that carry dust, odorants, and even the geosmin-producing bacteria themselves, sometimes over many kilometres. The question of whether also bees exploit this information should be followed up. Understanding the role of geosmin and a possible involvement in signalling imminent rain could also help to anticipate problems in the adaptation of bees to climate change and thus prevent ecological and economic damage.

## Methods

### Behavioural experiments

#### Honey bees and preparation procedure

Honey bee foragers were collected from various colonies of *Apis mellifera ligustica*, located in Rovereto, Italy from September 2019 to November 2019 and July 2020 to August 2020. The colonies were freely foraging and underwent routine beekeeping inspections during the entire period of the experiments. An equal number of bees from different colonies were included in the behavioural experiments. The bees were caught on sunny and cloudy days (but not on rainy days) in two rounds (around 10:30 AM or 14:00 PM).

Foragers were collected using a plastic container as they exited the hives, and they were brought back inside the lab and placed in an icebox. When the bees were motionless, they were placed in pairs into 50 ml centrifuge tubes modified into syringes. Two droplets of sucrose solution (50% sucrose water, vol/vol) were placed into the tube after the bees recovered completely. All honey bees were allowed to recover for at least 15 min (up to ~ 1 h for the last bees) before being tested in the set-up investigating stinging behaviour. If one or both bees showed signs of poor recovery when put in the setup (difficulty to hold upside down, disorientation and/or lethargic walk) the whole trial was excluded from further analysis. All the materials used to contain the bees were washed and cleaned with 80% ethanol, before the next use.

In total, 288 bees participated in the behavioural experiments, equally distributed between the 6 odour conditions (hence a sample size of 48 bees per group). This sample size was chosen based on previous studies^19,59^.

#### Odour stimuli

All odours were obtained from Sigma-Aldrich (98–99.9% purity) and were stored at 4 °C. They were diluted in mineral oil at the start of the experimental period and kept for the whole length of the behavioural experiment. When not in use these odours were sealed and stored at room temperature. The main component of the sting alarm pheromone, isoamyl acetate (IAA) was diluted to 10^–1^ (vol/vol) as in previous studies^19,59^. The concentrations of 10^–3^ and 10^–6^ were chosen for geosmin as these were shown to elicit behavioural responses in fruit flies^8^ and mosquitoes^10^.

The odours were delivered at room temperature (24 °C) by placing a filter paper soaked with 10 μl of odorant solution into an airflow that was injected via 3 equally spaced channels horizontally into the testing arena (Fig. 1a). The odours were removed from the arena via 40 equally spaced holes in the upper lid. The flow remained on during the whole duration of the trial (3 min). For testing interactions between 2 odours, 2 filter papers each carrying one of the odours were placed into the airflow. The incoming odour flow is controlled via a photoionization detector (200B miniPID, aurora scientific). However, this allows to resolve only the highest odour concentrations and serves to ensure the reproducibility of the conditions between experimental sessions. In the arena, the turbulent air motion inside unavoidably causes concentration fluctuations in space and time.

#### Stinging assay

The bees’ stinging responsiveness was tested using the assay described in detail in^19^. Briefly, dyads of honey bees were introduced into a cylindrical testing arena in which they confronted a black rotating dummy, prolonged by a black feather (Fig. 1a, Supplementary Movie 1). The primary function of the black feather is to disturb the bees without causing any pain, by brushing the sides of the arena. Note that the bees can easily avoid both dummy and feather. A trial lasted 3 min and was scored as “stinging” if at least one of the bees decided to sting the dummy during this time. This behaviour was defined as the bee holding onto the dummy or the feather for at least 3 s, with the tip of the abdomen pressed against it in the characteristic stinging posture. All the behavioural trials were recorded with a web camera (Microsoft Life cam) placed above the arena.

Two identical arenas and 2 dummies were used. Their use was balanced across the different odour conditions, to ensure that they did not contribute to potential differences in behaviour. Before each trial, the arena and the dummies with their feathers were cleaned using an 80% ethanol solution.

#### Data analysis

A generalized linear model (glm) was used to analyse the percentage of stinging trials. The odour group was set as a fixed factor while the hive and dummy were defined as random factors. A pairwise comparison (glht package in R) was done followed by Benjamini and Hochberg false discovery rate (FDR) correction for controlling Type I errors.

### Electroantennography (EAG)

#### Preparation procedure

The EAG technique was adapted from^60^, and the recordings were performed using a standard EAG apparatus (Syntech, Hilversum). Honey bee foragers were collected and handled in the same way as for the behavioural assay. After chilling them on ice, one antenna of each animal was cut at the level of the scape. A total of *n* = 24 antennae from both left and right sides were used for this experiment (14 left and 10 right antennae) to avoid lateralization effects^20^. The base of each antenna was then inserted into the glass reference electrode filled with Kaissling saline solution^61^ consisting of the following: NaCl (7.5 g/l); CaCl_2_ (0.21 g/l); KCl (0.35 g/l); NaHCO_3_ (0.2 g/l). The recording electrode was brought into contact with the last segment of the flagellum from which the distal tip had been cut. The recorded voltage drop elicited by an odour stimulus sums the depolarizations in all activated olfactory receptor neurons and depends on the number of activated neurons as well as their activity amplitudes.

#### Odour stimuli

A custom-made olfactometer was used to deliver odours to the bee antennae. The odour stimuli originate from glass vials containing 1 mL of odours dissolved in mineral oil. The olfactometer was operated using LabView and the single channels were switched by solenoid valves (LHDA0531115, The Lee Company) controlled by a PCIe-6321 multifunction board (National Instruments). The airflow during the recording is maintained constant during all phases of the experiment (For details see^62^).

Seven mineral oil solutions of either Geosmin (Geo) at concentrations 10^–6^, 10^–5^, 10^–4^, 10^–3^ vol/vol, Isoamyl Acetate (IAA) at concentrations 10^–3^, 10^–1^ vol/vol, or pure mineral oil (control) were prepared. Olfactometer glass vials were filled with these solutions. The stimulation protocol for each bee consisted of presenting each odour and a combination of odours in ascending order of concentrations, giving rise to 15 stimuli, see Fig. 2b–d: Control, Geo 10^–6^, Geo 10^–5^, Geo 10^–4^, Geo 10^–3^, IAA 10^–3^, IAA 10^–3^ + Geo 10^–6^, IAA 10^–3^ + Geo 10^–5^, IAA 10^–3^ + Geo 10^–4^, IAA 10^–3^ + Geo 10^–3^, IAA 10^–1^, IAA 10^–1^ + Geo 10^–6^, IAA 10^–1^ + Geo 10^–5^, IAA 10^–1^ + Geo 10^–4^, IAA 10^–1^ + Geo 10^–3^. The stimulus duration was 2 s and the inter-stimulus interval was 20 s. In addition, each cycle was repeated 10 times and therefore a total of 150 stimuli were presented to each bee antenna, this resulted in recordings of 55 min total duration. The odour flow reaching the antennae is monitored via a photoionization detector (200B miniPID, aurora scientific). However, this only allows to resolve odour concentrations > 10^–5^ and serves to ensure the reproducibility between single experimental sessions and between EAG and calcium imaging, because only for these experiments we can guarantee a uniform flow through the experimental area.

#### Data analysis

The response amplitude was calculated by subtracting the voltage averaged over 1 s before each stimulus from the voltage averaged over 1 s after stimulus onset. These values were thereafter averaged over the ten repetitions of each stimulus. Responses were analysed via repeated-measures ANOVA followed by Bonferroni post hoc tests.

Normalizations were done for each concentration of IAA (0, 10^–3^ and 10^–1^), by dividing the responses obtained for each concentration of geosmin combined respectively with each concentration by the response to the stimulus without geosmin. In the case of the control (MO), 1 was added to all the values before the normalization, preventing values between 0 and 1 in the normalization factor. After normalization, non-parametric tests (Friedman test followed by Dunn’s multiple comparisons), were performed for statistical comparisons.

### In vivo calcium imaging

#### Preparation procedure

Honey bees were prepared for the in vivo calcium imaging experiment according to a well-established protocol^62^. The foragers used were collected and immobilized in a fridge at 4 °C for 5–6 min. The immobilized bees were then fixed onto a custom-made imaging stage, using soft dental wax (Deiberit 502, Siladent). A small rectangular window was cut into the head cuticula of the fixed bee. The glands and trachea were moved aside and fura2-dextran, a calcium-sensitive fluorescent dye (Thermo-Fischer Scientific) dissolved in distilled water was injected into the antenno-cerebralis tracts right below the α-lobe using a microtip^63^. After the injection, the cuticula was fixed in its original state using n-eicosane. The bees were stored in a dark, cool, and humid place for about 20 h to ensure that the calcium dye has diffused into the AL.

Just before the imaging session, the cuticular window, trachea, and glands were completely removed. A silicone adhesive (Kwik-Sil) was used to cover the open window on top of the bee’s head, and it was left to dry for a few minutes. The fluorescent signal in the antennal lobe was then imaged under a two-photon microscope.

#### Two-photon microscopy

The two-photon microscope (Ultima IV, Bruker) is based on an ultra-short pulsed laser (Mai Tai, Deep See HP, Spectra-Physics). The laser was tuned to 780 nm for fura2 excitation. All images were acquired with a water-immersion objective (10 $\times$, NA 0.3, Olympus). The fluorescence was collected in epi-configuration, selected by a dichroic mirror, and filtered with a band-pass filter centred at 525 nm and with a 70 nm bandwidth (Chroma Technology Corp). Finally, it was detected by a photomultiplier tube (Hamamatsu Photonics). Laser powers of about 10 mW were used in order to balance signal-to-noise ratio (SNR) against photo-damage effects that reduced the bee life span.

The field of view of 280 × 280  µm^2^ was resolved by 128 × 128 pixels. The fluorescence intensity was recorded with a depth of 13 bits. The image acquisition at a frame rate of 10.1 Hz was synchronized to the stimulus protocol.

In addition to the functional images, a *z*-stack of the antennal lobe was acquired with a spatial resolution of 512 × 512 pixels and a z-layer distance of 2 µm to perform the morphological identification of glomeruli.

#### Odour stimuli

The olfactometer used to deliver odours under the two-photon microscope was the same one used in the EAG experiment. During an imaging session, the odorants of interest (Geosmin 10^–6^, Geosmin 10^–3^, and 10^–1^ IAA) were presented to the bee in a sequence either as a single odour or as mixtures, and the sequence was repeated 10 times. Each stimulus pulse lasted 3 s with a 12 s inter-stimulus interval and an exhaust system quickly removes the odours from the experimental area. For comparison of response strength and width also 3 floral odours were tested with the same sequences (1-nonanol 5·10^–3^, acetophenone 5·10^–3^, and 3-hexanol 5·10^–3^).

#### Data post-processing and analysis

Signals from a total of 14 bees were recorded and analysed. Data analysis was fully automated, based on custom MATLAB (R2019b, MathWorks) scripts. The fluorescence time series, containing an entire experimental sequence, were separated into periods of 3 s pre-stimulus, 3 s during the stimulus and 3 s post-stimulus for each trial. For each frame, we computed the relative fluorescence change

$$\Delta F/F (t)=-\frac{F\left(t\right)-{F}_{b}}{{F}_{b}},$$

via normalizing the raw fluorescence signal $F\left(t\right)$ by the average signal during the pre-stimulus period *F*_*b*_. This signal is proportional to the relative change in calcium concentration and thus the neuronal firing rate^64^. Δ*F*/*F* was averaged over the 10 trials for each odour. Next, the 2D activity maps were segmented for individual glomerular responses. The glomerular boundaries were obtained by recursively comparing three sources of information, the anatomical features from an additionally recorded 3D image stack (Supplementary Fig. 2a), the functional response maps (Supplementary Movie 2) and a regional homogeneity analysis that tests the correlation between the signals of each pixel and those from neighbouring pixels. This measure is high within individual glomeruli and falls off at their borders (Supplementary Fig. 2b). After coherently responding structures were segmented, the glomerular identity was determined using the digital 3D antennal lobe atlas 23. The analysis was limited to the 19 most frequently identified glomeruli. If the identity of individual glomeruli could not be determined with certainty, they were discarded, so that the total number of analysed glomeruli fluctuated across bees (Fig. 4c).

A statistical analysis of the subjects’ mean responses to single odours was performed via paired *t*-tests with FDR correction.

### Computational model

The neurons in the model are described by an adaptive leaky integrate-and-fire neuron with a membrane potential equation

$$C\frac{dV}{dt}=-{g}_{\text{leak}}\left(V-{V}_{\text{leak}}\right)-{g}_{\text{adapt}}a\left(V-{V}_{\text{adapt}}\right)+k{I}_{\text{ext}}+A\sigma ,$$

where $\sigma \sim \mathcal{N}\left(\mathrm{0,1}\right)$ is normally distributed white noise and the factor $k$ is used to rescale input currents in the ORNs. For olfactory receptor neurons (ORNs) the input current is the current passing through olfactory receptors (ORs) and $k=10$, while for local (LN) and projection neurons (PN), it is the synaptic currents from incoming synapses with $k=1$. All neurons have $C=1$ nF, ${V}_{\text{leak}}=-60$ mV, ${g}_{\text{leak}}=10$ nS, and $A=1.4$ nA. Whenever the membrane potential $V$ crosses the firing threshold ${V}_{th}=-40$ mV, a spike is emitted and the membrane potential is reset to ${V}_{\text{reset}}=-70$ mV. PNs do not have an adaptation current, but ORNs and LNs have spike rate adaptation with ${g}_{\text{adapt}}=1.5$ nS for ORNs, and ${g}_{\text{adapt}}=0.5$ nS for LNs. The adaptation variable was governed by

$$\frac{da}{dt}=0.5\sum_{{t}_{\text{spike}}}\delta \left({t}_{\text{spike}}\right)-\frac{a}{{\tau }_{\text{adapt}}},$$

where $\delta$ is the Dirac delta distribution and ${\tau }_{\text{adapt}}=1$ s for both ORNs and LNs.

Synapses are described with an instantaneous rise of synaptic activation $s$ upon arrival of a spike and subsequent exponential decay,

$$\frac{ds}{dt}=\sum_{{t}_{\text{spike}}}\delta \left({t}_{\text{spike}}\right)-\frac{1}{{\tau }_{\text{syn}}}s,$$

and a conductance-based input current into the post-synaptic neuron,

$${I}_{\text{ext}}={g}_{\text{syn}}s\left({V}_{\text{rev}}-V\right)$$

with reversal potential ${V}_{\text{rev}}=0$ mV for excitatory (ORN to PN, ORN to LN, PN to LN) and ${V}_{\text{rev}}=-80$ mV for inhibitory (LN to PN and LN to LN) synapses.

Olfactory receptors and the process of transduction are described by a standard two-stage binding and activation rate model, (see, e.g.^65–68^),

$${\dot{r}}_{0}=\sum_{j}{k}_{-1}^{j}{r}_{j}-\sum_{j}{\left({k}_{1}^{j}{c}_{j}\right)}^{n}{r}_{0}$$

$${\dot{r}}_{i}={\left({k}_{1}^{i}{c}_{i}\right)}^{n}{r}_{0}-{k}_{-1}^{i}{r}_{i}+{k}_{-2}^{i}{r}_{i}^{*}-{k}_{2}^{i}{r}_{i}$$

$${\dot{r}}_{i}^{*}={k}_{2}^{i}{r}_{i}-{k}_{-2}^{i}{r}_{i}^{*},$$

where ${r}_{0}$ is the fraction of unbound receptors, ${r}_{i}$ the fractions of receptors bound to odours $i=1, \dots , N$, and ${r}_{i}^{*}$ are the fractions of receptors bound to and activated by odours $i$. The constants ${k}_{1}^{i}$, and ${k}_{2}^{i}$ respectively describe the rate of binding to and being activated by odour $i$, while ${k}_{-1}^{i}$, and ${k}_{-2}^{i}$ describe the unbinding and inactivation. All $k$ constants can be specific to the odours and receptor types. In this work, we chose individual constants ${k}_{1}^{i}$ for each odour-receptor type pair and odour-specific (but equal for all receptor types) ${k}_{2}^{i}$. ${k}_{-1}^{i}={k}_{-2}^{i}=0.025$ kHz were identical for all odours and receptor types.

The individual binding constants for each odorant across receptors were chosen as Gaussian profiles,

$${k}_{1}^{i}\left(j\right)={10}^{{\eta }^{i}}{e}^{-\frac{{\pi \left(j\right)}^{2}}{2{\left({\sigma }^{i}\right)}^{2}}},j=1,\dots ,{N}_{\text{glo}},$$

where $\pi \left(\cdot \right)$ is a (randomly chosen) permutation of $1,\dots ,{N}_{\text{glo}}$, and $\eta$ is a ${\mathcal{N}}_{1.5,{0.5}^{2}}$ distributed random variable, truncated to values within $\left[0, 4\right]$. The standard deviation $\sigma$ of the odour profiles was sampled from ${\mathcal{N}}_{3,{0.5}^{2}}$, truncated to values greater or equal 1.5, all in units of kHz.

The activation constant ${k}_{2}^{i}$ for each odour was sampled from ${\mathcal{N}}_{0.02,{ 0.02}^{2}}$, truncated to values within $\left[0.0028, 0.2\right]$, in units of kHz.

For the simulations in this work, we generated 98 odours according to these rules and two additional odours, which we identify with IAA and Geosmin. For “IAA” we assumed a comparatively low sensitivity of ${\eta }^{\text{IAA}}=0.8$, and a narrow response profile with ${\sigma }^{\text{IAA}}=3$. The activation rate was with ${k}_{2}^{\text{IAA}}=0.1$ very high. For “Geosmin” we assumed high sensitivity ${\eta }^{\text{geo}}=4.4$, and a broad response profile with ${\sigma }^{\text{geo}}=10$, while the activation rate was moderate, ${k}_{2}^{\text{geo}}=0.003$. The true values of these parameters are unknown and the values chosen here ultimately were picked so that “IAA” and “Geosmin” responses exhibited the properties (strong monotonically growing response and vanishing response with increasing concentration) which we aimed to demonstrate as being feasible within this type of model. We generated the “IAA” and “geosmin” odours with a fixed distance of 30 OR types from peak to peak before scrambling both with the same permutation. This ensures that the two odours have a specific amount of overlap with implications in particular for the interactions at the OR on the antenna, as evidenced through the ORN spike counts.

The model network is illustrated in Fig. 4a. We simulated 160 receptor types^69^. Witthöft^70^ reported 5776 placoid sensilla on a worker bee’s antenna, with 12–20 ORNs in each sensillum, so in the range of 69,312 and 115,520 ORNs total). We have modelled 10 ORNs in a single model neuron with 10 times higher firing rates for numerical efficiency, i.e. 60 ORN model neurons per type, 9600 in total. This is a common practice in olfactory systems modelling, which is exact where ORNs are modelled as Poisson processes, and a good approximation for other neuron model types. Note that ORNs are all assumed to not interact, which allows this simplification. The AL has an estimated 800 PNs^71,72^, which equates to 5 simulated PNs per glomerulus and 4000 LNs^70^, i.e*.* 25 simulated LNs per glomerulus. See also^23^ for a very similar published model. Each of the 5 PNs and 25 LNs in a glomerulus are connected to 12 randomly chosen ORNs of their receptor type. PNs excite all LNs in their glomerulus and LNs inhibit all PNs and LNs in all glomeruli except their own, implementing a commonly assumed network motif of all-to-all lateral inhibition. The synapse parameters for these connections are summarised in Table 1. All differential equations were integrated with a linear Euler algorithm and global 0.2 ms timestep.

**Table 1:** Synapse parameters.

	ORN $\to$ PN	ORN $\to$ LN	PN $\to$ LN	LN $\to$ PN	LN $\to$ LN
${g}_{\text{syn}}$ (nS)	8	8	1	0.055	0.02
${\tau }_{\text{syn}}$ (ms)	10	10	10	20	20
${V}_{\text{rev}}$ (mV)	0	0	0	− 80	− 80

The models were implemented using the PyGeNN interface^73^ for GeNN 4.5.0^74,75^. GeNN is available at https://github.com/genn-team/genn and the source code for the modelling work in this paper is available at https://github.com/tnowotny/bee_al_2021, including Jupyter notebooks for analysis and plotting. Simulations were run on a Linux workstation, running Ubuntu 18.04.5 LTS.

#### Monotonicity of odour responses in the model

The monotonicity of the response x (x = maximal spike density function (SDF) or averaged SDF, each across all glomeruli) in this context was calculated as.

$$m=\frac{x\left({10}^{-1}\right)-{\text{max}}\left(x\right)}{{\text{mean}}\left(x\right)},$$

i.e. the difference between the SDF at the highest tested odour concentration ($${10}^{-1})$$ and the highest observed SDF, normalised by the mean observed SDF, where the maximum and mean were taken across all concentrations from $${10}^{-7}$$ to $${10}^{-1}$$. Monotonicity takes values smaller or equal 0, and monotonic odours have $$m=0$$.

#### Spike density function

We calculated Spike Density Functions (SDF) as a smooth firing rate estimate^76^. The SDF was calculated by the convolution of the spike train (understood as a point process) with a Gaussian kernel function,

$${\text{SDF}}\left(t\right)=\frac{1}{\sigma \sqrt{2\pi }}\sum_{{t}_{\text{spike}}}{e}^{-\frac{{\left(t-{t}_{\text{spike}}\right)}^{2}}{2{\sigma }^{2}}}.$$

with $$\sigma =100$$ ms.

## Supplementary Information

Supplementary Information 1.

Supplementary Video 1.

## Data Availability

The datasets acquired and analysed during the current study are available from the corresponding author (albrecht.haase@unitn.it) on reasonable request, the source code for the modelling is available at https://github.com/tnowotny/bee_al_2021.
