# Supplementary material for: Geosmin suppresses defensive behaviour and elicits unusual neural responses in honey bees
Source: Sci Rep. 2023 Mar 8;13:3851. doi: 10.1038/s41598-023-30796-5 (PMC9995521; doi:10.1038/s41598-023-30796-5)
Supplement: Supplementary file 1 — Supplementary Information 1. [file 41598_2023_30796_MOESM1_ESM.docx]

**Geosmin suppresses defensive behaviour and elicits unusual neural responses in honey bees.**

**Supplementary material:**

**
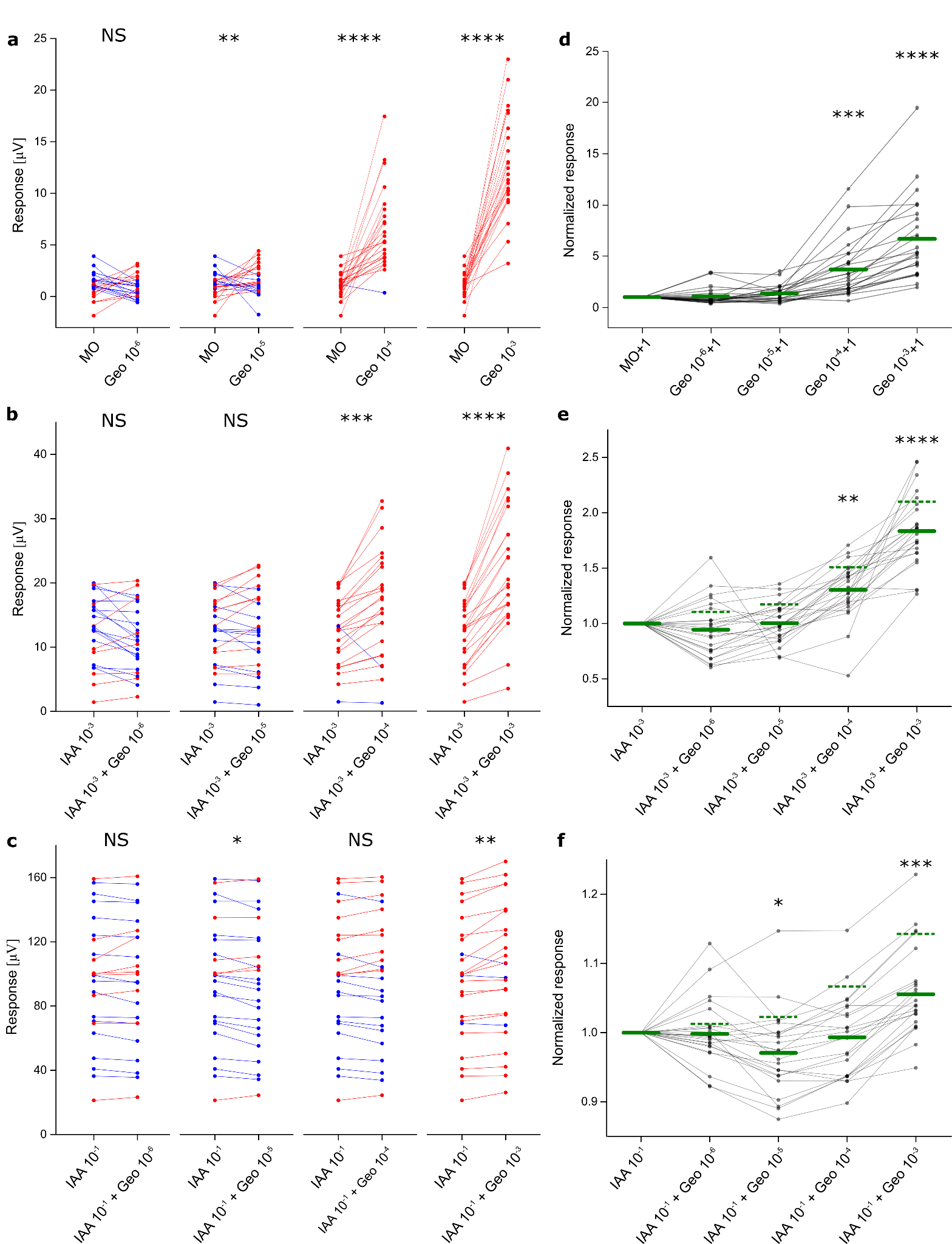
**

**Supplementary Fig 1. Individual bees EAG responses. a-c)** Voltage changes in response to different concentrations of IAA in presence or absence of geosmin. Individuals represented in red showed a bigger response amplitude in presence of geosmin than in its absence, while those represented in blue showed a comparative lower response when geosmin was present. Statistics are RM-ANOVA followed by Bonferroni multiple comparisons with Control, IAA3, and IAA1 respectively. **(d-f).** Normalised response amplitude for each individual, connected points correspond to responses from the same bee. Before the normalisation, 1 was added to all the values in (d), in order to prevent the normalising factor from having values between 0 and 1, which was the most frequent situation. Green continuous lines represent the mean normalised experimental response for each combination of odours. Green dashed lines denote the mean (normalised) expected activity from a purely additive interaction. Statistics are Friedman test followed by Dunn’s multiple comparisons with Control, IAA3, and IAA1 respectively. ****: *p* < 0.0001; ***: *p* < 0.001; **: *p* < 0.01; *: *p* < 0.05. MO: mineral oil (solvent) control; IAA 10^-^*^x^*: Isoamyl acetate 10^-^*^x^* (vol/vol); Geo 10*^-x^*: Geosmin 10^-^*^x^* (vol/vol);

**
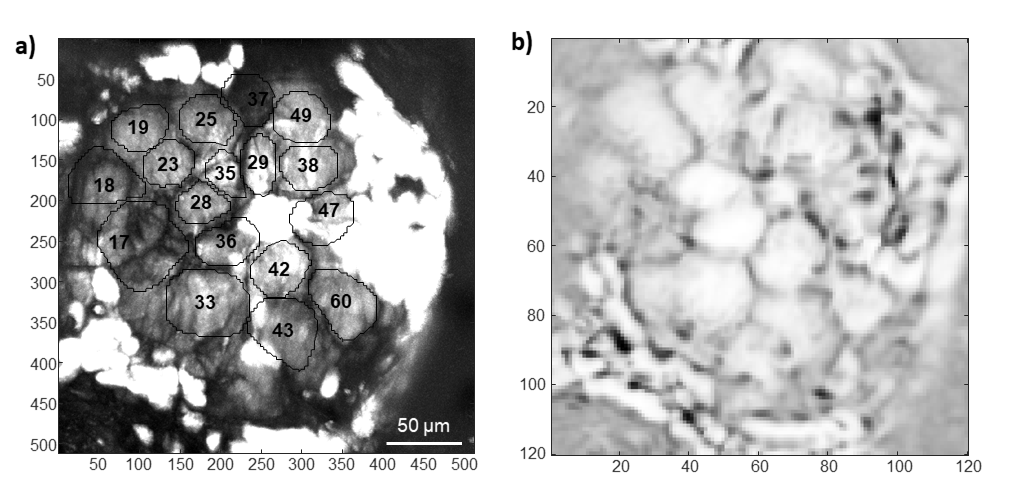
Supplementary Fig 2. Anatomical map of the antennal lobe**. **a)** Exemplary projection view on a single left antennal lobe. Superimposed are regions of interest, together with labels of glomeruli they are associated with. **b)** Regional homogeneity (ReHo) analysis of functional data, the grey value reflects the average correlation of each pixel with its neighbours across all odour stimuli. Glomerular boundaries are obtained by comparison of anatomical features, activity maps (Supplementary Movie 2), and the ReHo maps. The identity of the 19 analysed glomeruli is determined via the digital 3D antennal lobe atlas [1].

**Supplementary Fig. 3**. **Odour responses in PNs as a function of concentration.** For a selection of 3 different properties of the odour profile: (i) the breadth $\sigma$of the odour profile, the overall sensitivity $\eta$of the ORs to the odour, and the activation *k_2_* of the ORs. The colourmaps all use a common colour scheme as illustrated by the colour bar. The panel with the red rectangle is the “geosmin” odour. Note how the non-monotonic responses start appearing for increasing breadth (from left to right) and how for very broad profiles the responses disappear.
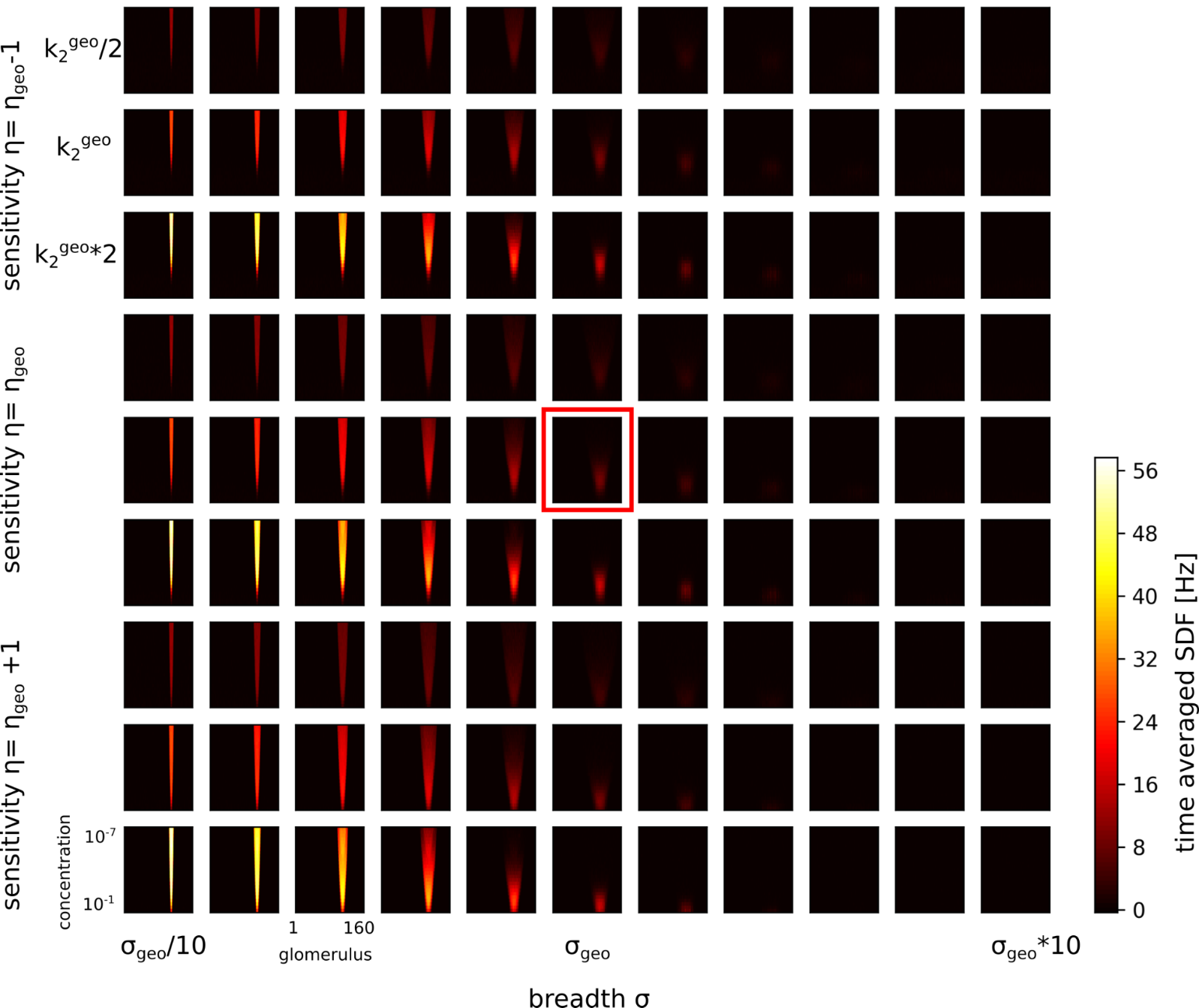


**Supplementary Fig. 4.** Same as Supplementary Fig. 2 but with individual colour scale for each sub-panel to reveal the details of the spiking activity.
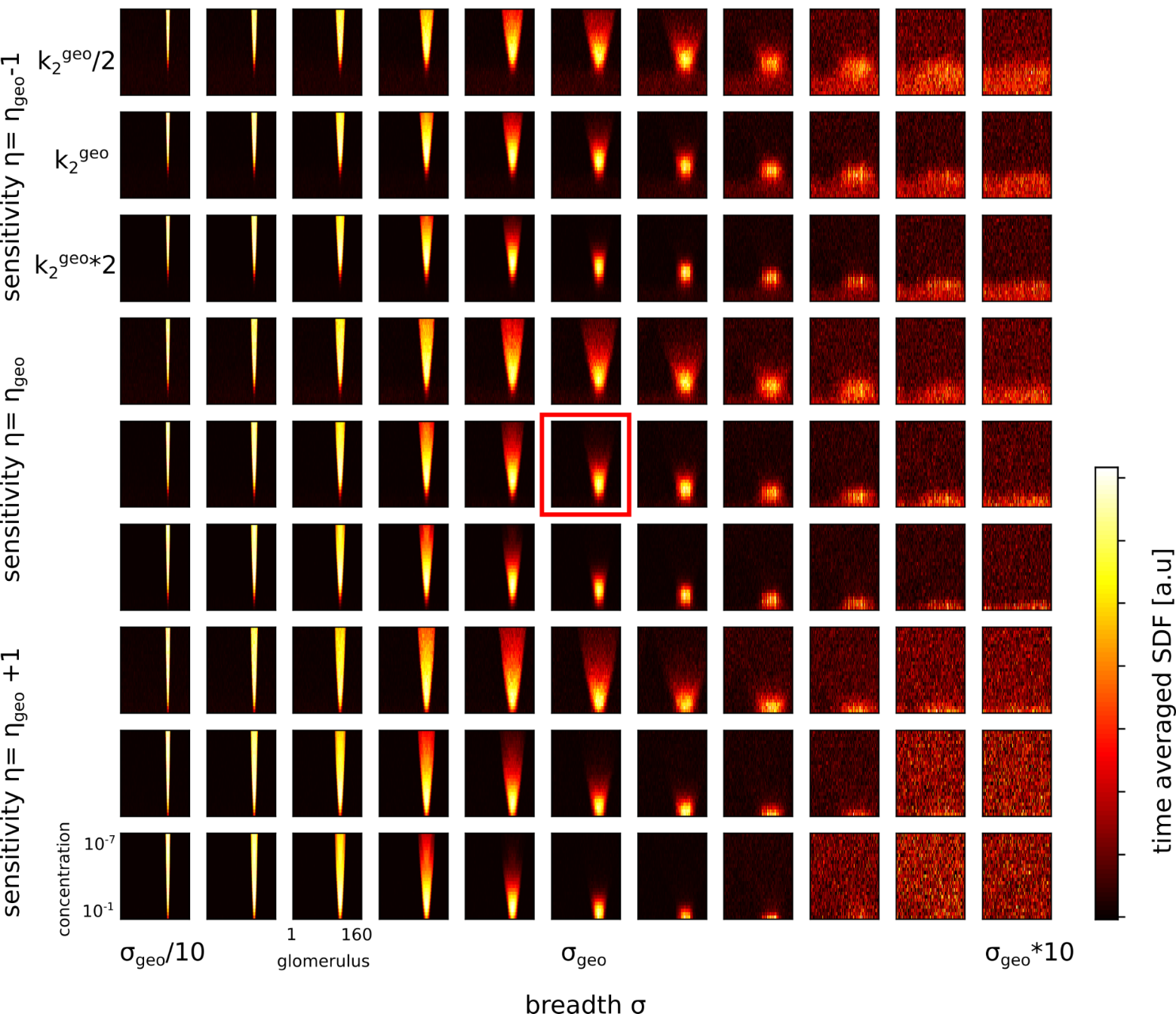


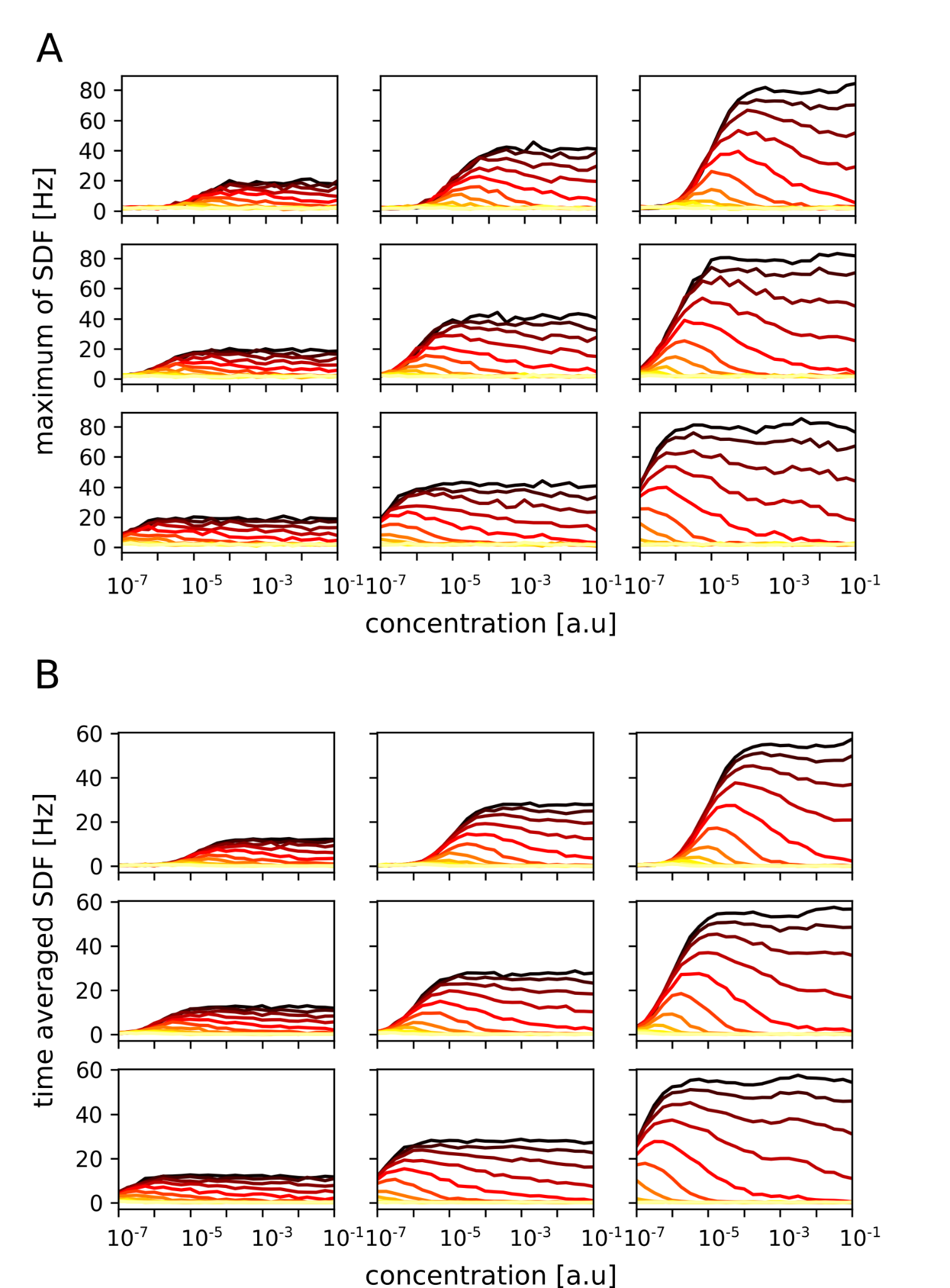


**Supplementary Fig. 5. Response of the strongest glomerulus.** Dependence of the response of the PNs of the strongest glomerulus as a function of concentration (*x*-axes). **A.** Maximal PN response in the most strongly responding glomerulus. **B.** Time-averaged response of the PNs in the most strongly responding glomerulus. The individual panels correspond to activation *k_2_^geo^*/2, *k_2_^geo^*, *k_2_^geo^*$*$2 (left to right) and sensitivity $\eta$*_geo_*-1, $\eta$*_geo_*, $\eta$*_geo_*+1 (top to bottom). The coloured lines are for increasing odour profile breadth $\sigma$ from $\sigma$*_geo_* /10 (black) to $\sigma$*_geo_*$*$10 (yellow).


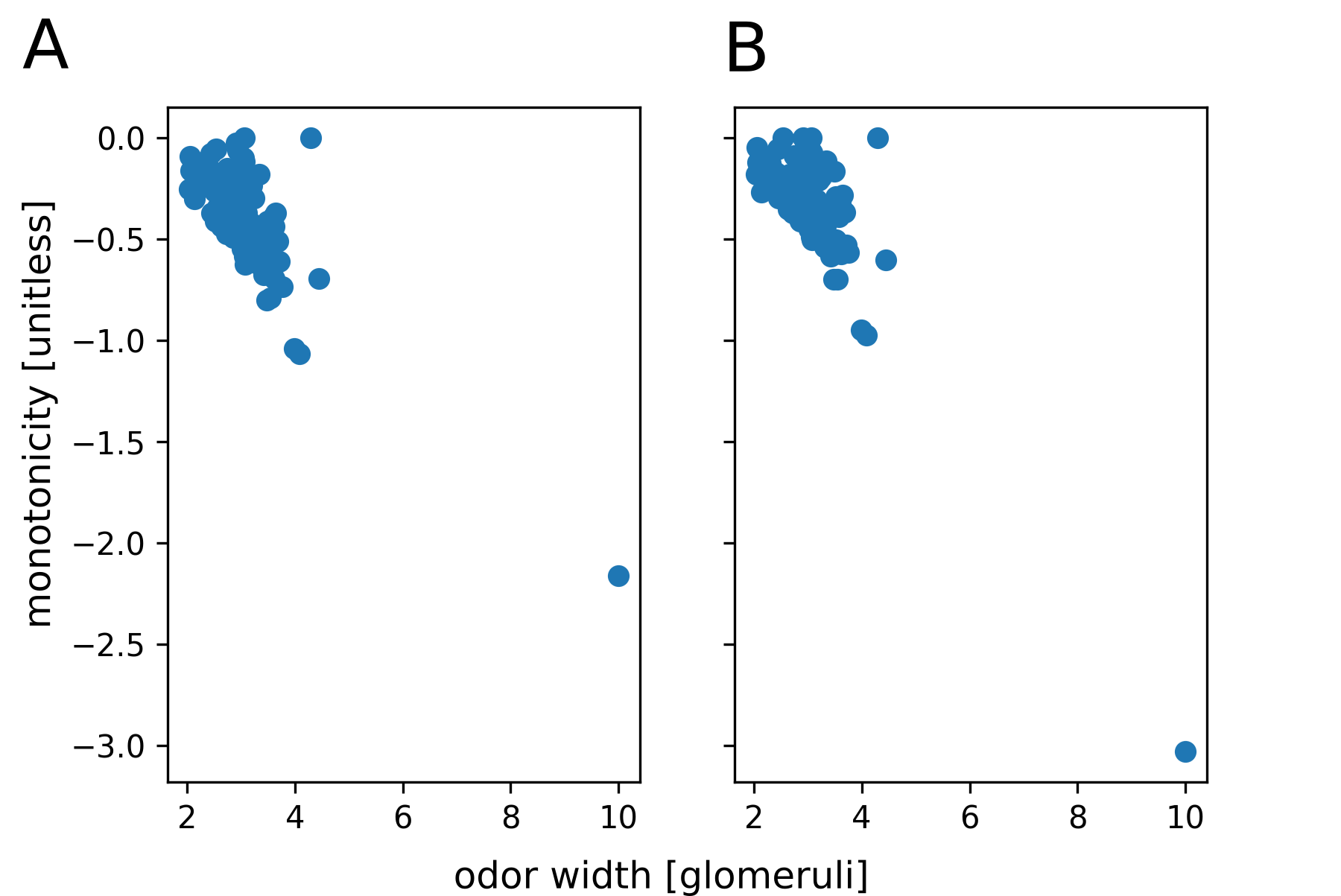


**Supplementary Fig. 6. Monotonicity-width relation.**  Relationship between the width of the odour profile and the observed monotonicity of the response of PNs. **A.** Monotonicity calculated with respect to the maximal (phasic) response and **B.** Monotonicity calculated with respect to the time-averaged response during odour presentation. The monotonicity of the response *x* (*x* = maximal spike density function (SDF) or averaged SDF) in this context was calculated as m = (*x*(10^-1^) - max(*x*))/mean(*x*), where the maximum and mean were taken across all concentrations from 10^-7^ to 10^-1^. Monotonicity takes values <= 0, where monotonic odours have *m* = 0. Monotonicity is strongly anti-correlated with odour profile width with *R* = -0.802 for the maximal SDF (A) and *R* = -0.862 for the average SDF (B).


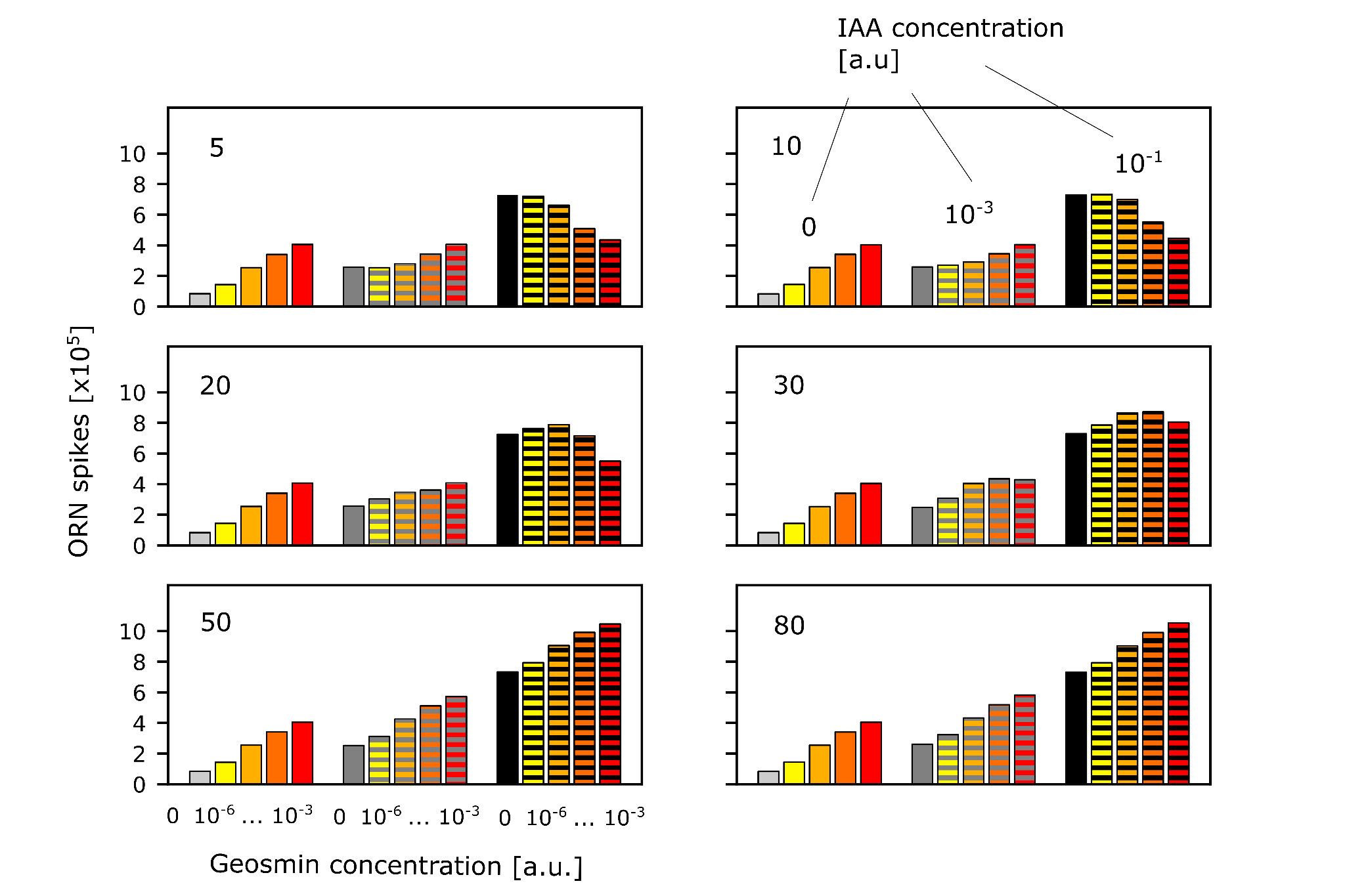


**Supplementary Fig. 7. Interaction of “geosmin” and “IAA” on the antenna for different similarities of their Gaussian odour profiles.** The numbers in the top left corner of each panel denote the distance (in units of glomeruli) of the Gaussian peaks describing the “geosmin” and “IAA” odours. For very small distances (very similar response profiles), there is a strong inhibitory interaction, whereas for large distances (very dissimilar response profiles) there is no visible suppression.

**Movie 1. Initial phase of the stinging assay.** A dyad of bees is inserted into the experimental arena and one of them immediately attacks the rotating dummy.

1. Galizia CG, McIlwrath SL, Menzel R. 1999 A digital three-dimensional atlas of the honeybee antennal lobe based on optical sections acquired by confocal microscopy. *Cell Tissue Res.* **295**, 383–394. (doi:10.1007/s004410051245)
